# MoMtg1 Acts as a Novel Transcriptional Repressor of MoSwi6 During Appressorium‐Mediated Penetration in the Rice Blast Fungus

**DOI:** 10.1002/advs.202509002

**Published:** 2025-08-28

**Authors:** Xingyu Wang, Ting Zhang, Qi Li, Chang‐An Ji, Lin Huang, Mingzhi Zhang, Leiyun Yang, Xinyu Liu, Muxing Liu, Gang Li, Zhengguang Zhang, Haifeng Zhang

**Affiliations:** ^1^ State Key Laboratory of Agricultural and Forestry Biosecurity College of Plant Protection Nanjing Agricultural University Nanjing 210095 China; ^2^ College of Forestry and Co‐Innovation Center for Sustainable Forestry in Southern China Nanjing Forestry University Nanjing 210037 China; ^3^ Jiangsu Key Laboratory of Pesticide Science College of Sciences Nanjing Agricultural University Nanjing 210095 China

**Keywords:** appressorium cell cycle, disease control target, MoMtg1, transcription factor MoSwi6, transcriptional repressor

## Abstract

Appressoria are specialized penetration structures for many plant pathogenic fungi, including the rice blast fungus *Magnaporthe oryzae*, which evolves a set of complicated regulatory mechanisms to control appressorium development and function. Cell cycle control is essential for appressorium‐mediated penetration, but the mechanism underlying its role remains largely elusive. Here, a conserved protein MoMtg1 is identified in filamentous fungi as a novel transcriptional repressor that plays a crucial role in cell cycle regulation. MoMtg1 directly interacts with transcription factor MoSwi6 and inhibits its transcriptional activity. Deletion of MoMtg1 or MoSwi6 results in cell cycle defects during appressorium development. Both mutants are abnormal in melanization, appressorium turgor generation, reactive oxygen species (ROS) accumulation, and septin assembly. MoSwi6 positively and MoMtg1 negatively regulate the expression of *MoCYC1*, the cyclin gene essential for maintaining normal appressorium development. Overexpression of *MoCYC1* in the wild type resulted in similar defects in appressorium development and function with ∆*Momtg1* and ∆*Moswi6* mutants. It is also shown that silencing *MoMTG1* with the host‐induced gene silencing strategy conferred resistance against *M. oryzae* in transgenic plants. Furthermore, a small molecule is identified as a MoMtg1‐inhibitor by protein modelling and shows to inhibit MoMtg1 functions and reduce *M. oryzae* infection. Overall, the study reports a novel cell cycle regulator and its underlying mechanisms during appressorium‐mediated penetration, which has great potentials as a target for disease control.

## Graphical abstract

Appressoria are specialized penetration structures for many plant pathogenic fungi, demanding precise cell cycle control. This study unveils MoMtg1 as a transcriptional repressor of MoSwi6, orchestrating *MoCYC1* expression during turgor‐driven cell cycle progression to enable appressorium‐mediated penetration. Moreover, a small‐molecule inhibitor ZS1619 targeting MoMtg1 effectively controls the rice blast disease.

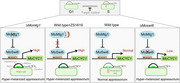

## 1 Introduction

The cuticle of plants serves as a vital barrier to pathogen invasion. To gain entry to host tissues, many fungal pathogens develop specialized infection structure called appressoria for penetration. Appressoria are essential to plant infection in a number of important plant pathogenic fungi that cause devastating diseases such as corn anthracnose, corn smut and potato late blight.^[^ ^1^, ^2^ ^]^ Similar to many other phytopathogens, the rice blast fungus, responsible for the most destructive disease in cultivated rice, depends on appressoria to penetrate the rice surface.^[^ ^3^, ^4^, ^5^ ^]^ Inhibiting appressorium formation and penetration offers one of the most effective strategies to control the rice blast disease.

When germinating conidia recognize the physical and chemical signals on the host, the tip of germ tubes develops into a dome‐shaped cell.^[^ ^6^, ^7^ ^]^ All the contents of conidia are degraded by autophagy pathway and then transported into appressoria for glycerol accumulation and generation of extremely higher turgor pressure.^[^ ^8^, ^9^ ^]^ To maintain the turgor, a melanin layer is deposited between the cell wall and cytoplasm membrane in appressoria to prevent the effusion of internal contents.^[^ ^10^ ^]^ When cellular turgor achieves a critical threshold, the sensor kinase Sln1 activates the NADPH oxidases (Nox) complex, which is necessary for regulating the synthesis of ROS. The ROS are required for septin‐mediated cytoskeletal reorganization. The septin GTPases are recruited to form a ring structure at the appressorium pore, providing a scaffold for filamentous actin (F‐actin) ring remodeling.^[^ ^11^, ^12^, ^13^ ^]^ Correctly assembled F‐actin triggers the repolarization process in appressoria, leading to the formation of penetration pegs at the appressorium pore. After breaching plant epidermal cells, the penetration peg further differentiates into invasive hyphae and establishes colonization.^[^ ^14^, ^15^, ^16^ ^]^

Appressorium morphogenesis and penetration peg formation involve two rounds of mitosis, which is strictly regulated by the completion of deoxyribonucleic acid (DNA) replication or the synthesis phase (S‐phase) of the cell cycle.^[^ ^17^ ^]^ Two independent S‐phase checkpoints operate in these two consecutive cell cycles. The first S‐phase checkpoint is activated by the DNA damage response (DDR) pathway during conidial germination. Disruption of the S‐phase checkpoint, either by hydroxyurea (HU) treatment or temperature‐sensitive mutations in Nim1, results in the failure of germ tubes to develop into appressoria.^[^ ^18^, ^19^ ^]^ The second S‐phase checkpoint occurs when turgor reaches a proper level, melanin biosynthesis and glycerol accumulation are inhibited, triggering the proper assembly of the F‐actin ring followed by penetration peg development. Disruption of this S‐phase checkpoint leads to hyper‐melanized appressoria with increased turgor, resulting in a complete loss of infectivity. This process depends on the activity of B‐type cyclin gene *MoCYC1*. The temperature‐sensitive mutant *Mocyc1^nimE10^* fails to form penetration peg and infect rice tissues.^[^ ^17^ ^]^ Notably, its function differs from that of cyclin B proteins in other eukaryotic cells. In human cells, the proper expression level of cyclin B is essential for the S/G2 transition. Unregulated accumulation of cyclin B can lead to aberrant DNA replication during the S phase, potentially causing cell cycle disorder.^[^ ^20^ ^]^ The sensor kinase Sln1 plays a critical role in S phase of the second cell cycle. The absence of Sln1 also leads to defects in appressorium melanization and turgor generation and loss of infectivity.^[^ ^13^ ^]^ However, the number of cell cycle‐related genes characterized in the rice blast fungus is limited and regulatory mechanisms of cell cycle during appressorium formation remain to be characterized.

In this study, we identified MoMtg1 as a novel protein that plays a crucial role in cell cycle and appressorium mediated penetration of *M. oryzae*. Whereas the transcription factor MoSwi6 directly activates the expression of the B‐type cyclin gene *MoCYC1* in G1/S phase of the second cell cycle, MoMtg1 acts as a transcription repressor that interacts with MoSwi6 to control the normal expression of *MoCYC1*, ensuring the progression of the cell cycle during appressorium maturation. Importantly, the absence of MoMtg1 in animals and plants highlights its potential as a target for host induced gene silencing (HIGS) and novel fungicide design to control rice blast disease.

## 2 Results

### 2.1 MoMtg1 is Important for Vegetative Growth, Conidiogenesis, and Penetration

**Figure 1:**
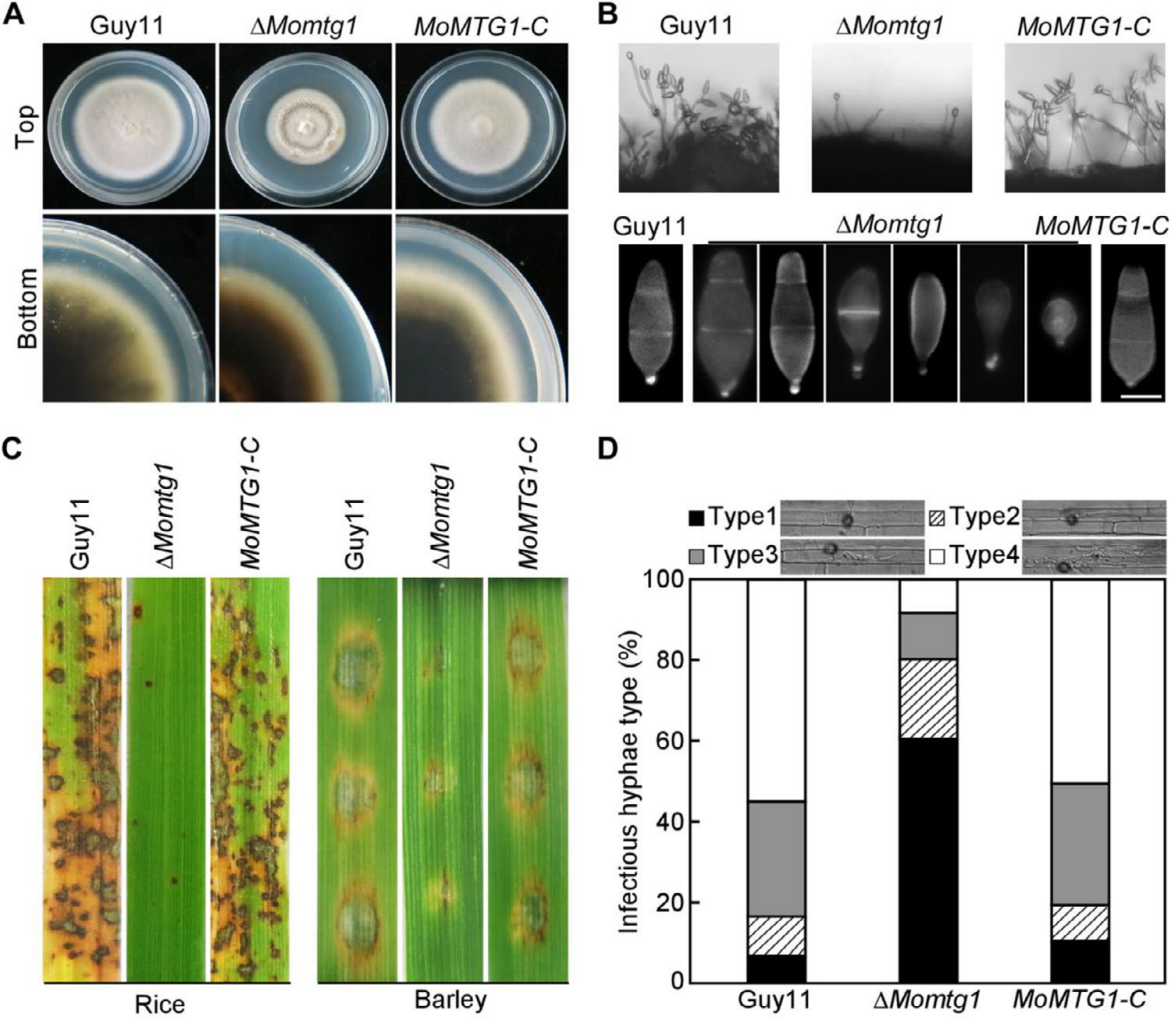
MoMtg1 is important for vegetative growth, conidiogenesis and penetration. A) The wild‐type Guy11, Δ*Momtg1* mutant and the complemented transformant *MoMTG1*‐*C* were cultured on CM medium, and photographed at 7 days after incubation (dai). B) Conidial development was induced on glass slides and examined under a light microscope at 20 h post‐inoculation (hpi), and conidial morphology was observed under a differential interference contrast (DIC) microscope. C) Conidial suspensions of the indicated strains were sprayed onto susceptible rice seedlings or dropped onto detached barley leaves, and photographed at 7 (rice) or 5 dai (barley). D) Conidial suspensions were injected into detached rice sheaths and the invasive hyphae (IH) were examined and statistically analyzed at 32 h post‐inoculation (hpi). IH type: type 1, no penetration; type 2, with penetration peg; type 3, with secondary invasive hyphae restricted in one cell; type 4, with extensive hyphal growth expanding to neighboring cells.

Our previous studies reported that the transcription factor MoMsn2 plays pleiotropic roles in *M. oryzae* by regulating the expression of various genes, including MGG_03546, a novel gene with no functional domain.^[^ ^21^, ^22^ ^]^ In this study, MGG_03546 was identified a target of MoMsn2 and named *MoMTG1* (MoMsn2‐target‐gene) as MoMsn2 positively regulates its expression via binding to its promoter region, (Figure S1A–D, Supporting Information). To investigate the biological role of MoMtg1 in *M. oryzae*, we generated the *MoMTG1* gene deletion mutant ∆*Momtg1* by gene replacement strategy and confirmed the mutant by Southern blot analysis (Figure S2A,B, Supporting Information). Compared to the wild type Guy11 and the complemented transformant *MoMTG1‐C*, ∆*Momtg1* formed smaller colonies with reduced aerial hyphae and it was defective in pigmentation as well as produced a lower number of conidia with abnormal morphology (**Figure** **1**A,B; Table S1, Supporting Information). The ∆*Momtg1* mutant was defective in penetration and infectious growth (Figure 1C,D) but it was normal in conidium germination and appressorium formation (Table S1, Supporting Information). These results suggest that MoMtg1 plays a crucial role in vegetative growth, conidiogenesis and pathogenesis in *M. oryzae*.

### 2.2 Deletion of MoMtg1 Leads to Defects in Appressorium, Melanization, and Turgor

**Figure 2:**
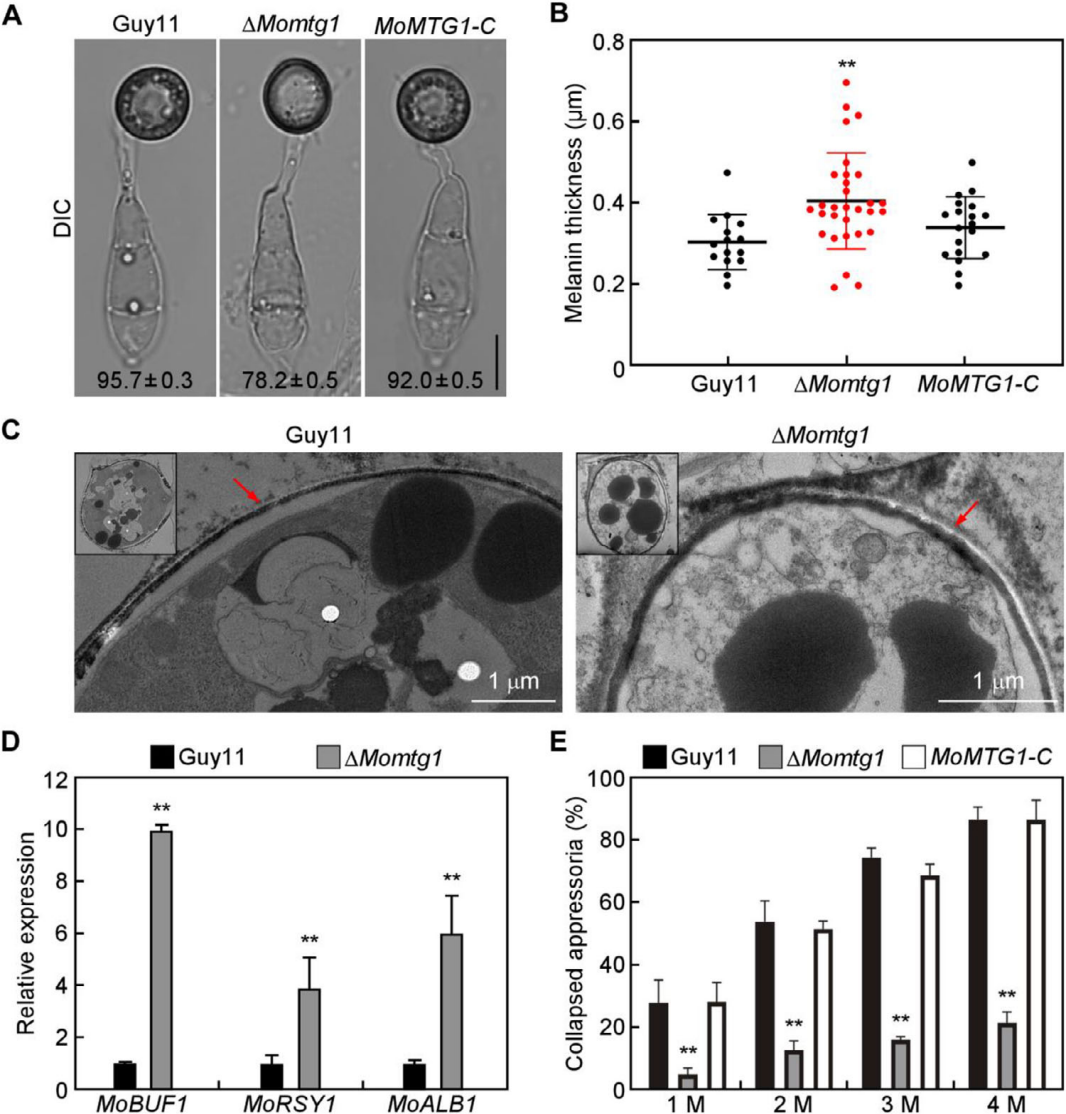
Deletion of MoMtg1 leads to hyper‐melanized appressoria with increased turgor. A) Appressoria formed by conidia of the indicated strains was observed after incubation on hydrophobic cover glasses for 24 h. The numbers represent the percentage of conidia formed melanized appressoria. Mean and standard deviation (SD) were calculated from three independent experiments with 100 conidia examined in each replicate. Bar=10 µm. B) Statistical analysis of the appressorium melanin thickness (*n*=3 experiments, appressoria=15–30). Asterisks indicate significant differences (*p* < 0.01, two‐tailed Student's *t*‐test). C) Melanin layers in appressoria of Guy11 and Δ*Momtg1* were examined under TEM (Hitachi HT‐7800 80KV). Red arrows indicate melanin layers. D) Assays of the expression levels of three melanin‐biosynthesis genes by qRT‐PCR. Error bars are standard deviations from three biological replicates, and asterisks indicate significant differences (*p* < 0.01, two‐tailed Student's *t*‐test). E) Appressorium turgor was determined by 1.0–4.0 m of glycerol solution. Error bars are standard deviations from three biological replicates, with 100 appressoria examined per replicate. Asterisks represent significant differences (*p* < 0.01, two‐tailed Student's *t*‐test).

The penetration defect of ∆*Momtg1* implicates its appressorium dysfunction. When appressorium morphology was examined, 78.2% of appressoria formed by ∆*Momtg1* exhibited a thicker melanin layer in comparison with those of Guy11 and *MoMTG1‐C* (**Figure** **2**A,B), which was confirmed by transmission electron microscopy (TEM) examination (Figure 2C). Quantitative RT‐PCR analysis revealed that the expression level of three melanin biosynthesis genes, *MoBUF1*, *MoRSY1* and *MoALB1*, was significantly increased in the appressoria of ∆*Momtg1* (Figure 2D). Furthermore, cytorrhysis assays revealed that the intracellular turgor of ∆*Momtg1* appressoria was much higher than those of Guy11 and *MoMTG1‐C* (Figure 2E). These results suggest that MoMtg1 is critical for proper appressorium melanin and turgor generation, which is essential for appressorium‐mediated penetration.

### 2.3 MoMtg1 is Important for Proper ROS Production and Septin Assembly During Appressorium Formation

Proper cellular turgor and the synthesis of ROS regulated by NADPH oxidases are required for septin organization at the appressorium pore.^[^ ^12^, ^23^ ^]^ Therefore, we detected ROS production during appressorium formation by staining with 2′,7′‐dichlorodihydrofluorescein diacetate (DCFH‐DA). A burst of ROS within appressoria of the wild type Guy11 was observed after incubation for 3–8 h but not at 12 or 24 h. However, ROS burst within *Momtg1* appressoria of was observed during the entire process of appressorium development (Figure S3A,B, Supporting Information). We subsequently investigated the septin organization at the appressorium pore by expressing the septin marker proteins MoSep4 and MoSep5 in Guy11 and ∆*Momtg1*. Our examination revealed that intact septin rings were observed in ≈80% of the appressoria formed by Guy11 but only 25% in ∆*Momtg1* (Figure S3C–D, Supporting Information). These results suggest that MoMtg1 is involved in the regulation of ROS production and septin assembly during appressorium formation.

### 2.4 MoMtg1 Plays A Crucial Role in Turgor‐Dependent S‐Phase Progression During Appressorium Maturation

**Figure 3:**
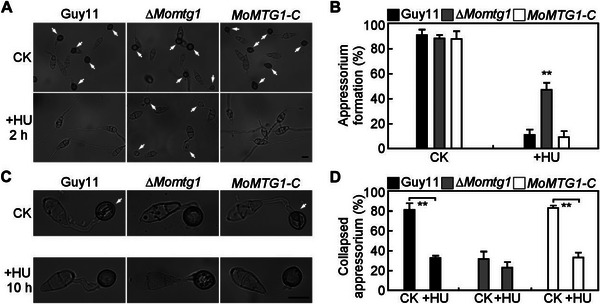
MoMtg1 plays a crucial role in turgor‐dependent S‐phase progression during appressorium maturation. A) Appressorium formation of the indicated strains exposure to 200 mm HU, added at 2 hpi and observed at 24 hpi. White arrows indicate appressoria. CK, ddH_2_O control. Bar=10 µm. B) Statistical analysis of the appressorium formation with or without HU treatment. Error bars are standard deviations from three biological replicates with 100 conidia counted per replicate, and asterisks represent significant differences (*p* < 0.01, two‐tailed Student's *t*‐test). C) Effect of 4 m glycerol to appressoria with or without 200 mm HU treatment at 10 hpi, and observed at 24 hpi. White arrows indicate collapsed appressoria. Bar=10 µm. D) Statistical analysis of the collapsed appressorium with or without HU treatment. Error bars are standard deviations from three biological replicates with 100 appressoria counted per replicate, and asterisks represent significant differences (*p* < 0.01, two‐tailed Student's *t*‐test).

Two independent S‐phase checkpoints have been identified to regulate appressorium morphogenesis and turgor‐dependent repolarization. Arrest in the second checkpoint increases appressorium melanin content and turgor generation.^[^ ^17^, ^18^, ^19^ ^]^ Because deletion of MoMtg1 leads to hyper‐melanized appressoria with increased turgor, suggesting that it likely plays a role in cell cycle control during appressorium formation. Therefore, we assayed the effect of the DNA replication inhibitor HU at initial appressorium development (2 hpi) and found that 48.3% ∆*Momtg1* conidia formed appressoria but only 10.2% in Guy11 (**Figure** **3**A,B). This result indicates that ∆*Momtg1* was able to bypass the first S‐phase arrest triggered by HU during initial appressorium morphogenesis. We then measured turgor pressure of the mature appressoria with HU treatment at 10 hpi, and found that ∆*Momtg1* exhibits similar higher turgor pressure in appressoria in the presence HU as the control. In contrast, the appressoria turgor of Guy11 with HU treatment at 10 hpi showed a significant increase, with 33.3% collapsed rate compared to 81.6% in the untreated control (Figure 3C,D). These results indicated that MoMtg1 is essential for successful S‐phase transition during appressorium morphogenesis.

### 2.5 MoMtg1 Interacts with MoSwi6 that is also Involved in Turgor‐Dependent S‐Phase Progression

**Figure 4:**
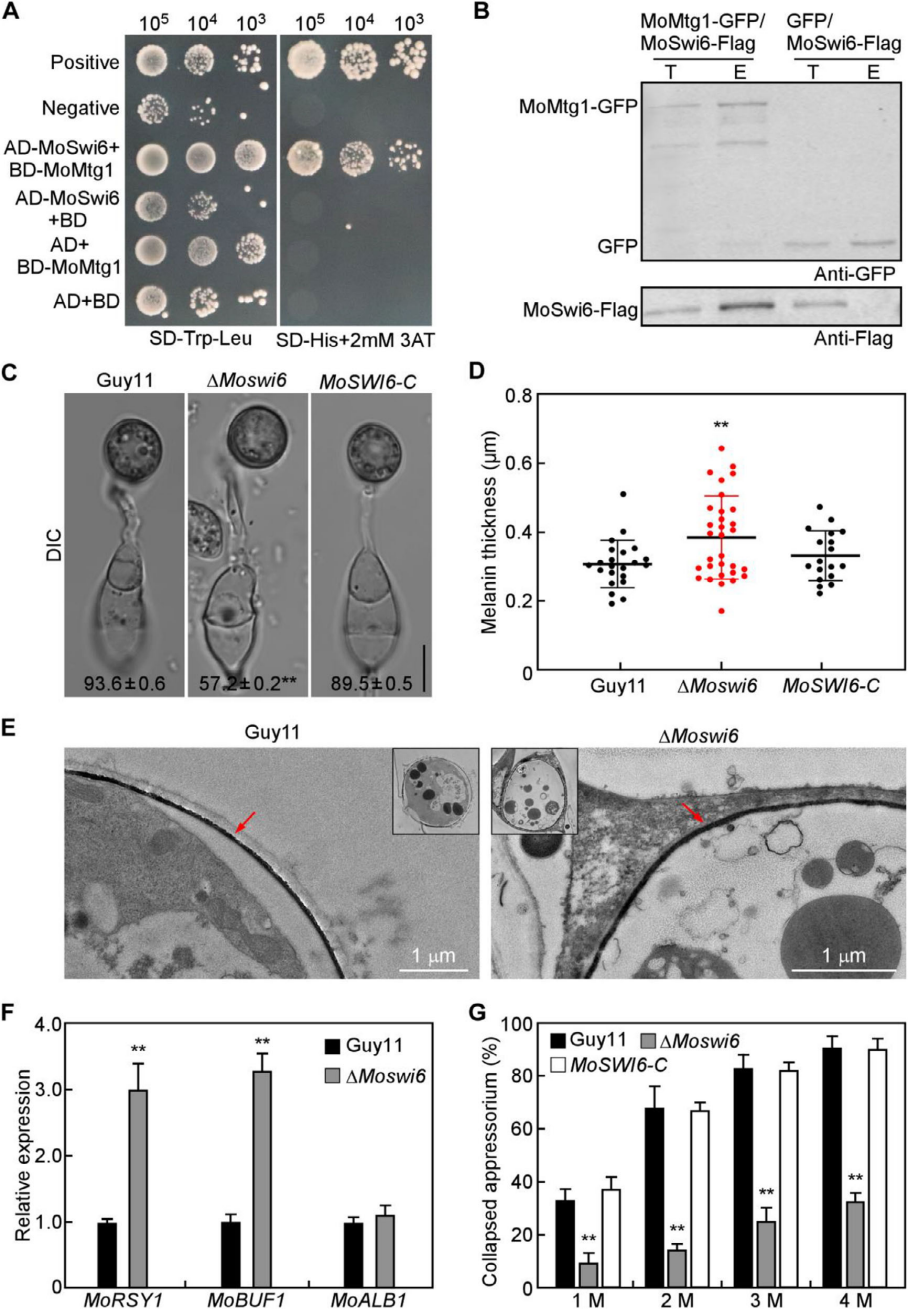
TF MoSwi6 interacts with MoMtg1 and also involves in turgor‐dependent S‐phase progression. A) Yeast two hybrid assay analyses the interaction between MoSwi6 and MoMtg1. B) Co‐IP assay analyses the interaction between MoSwi6 and MoMtg1 in appressorium stage. C) Appressoria formed by conidia of the indicated strains was observed after incubation on hydrophobic cover glasses for 24 h. The numbers represent the percentage of conidia formed melanized appressoria. Mean and standard deviation were calculated from three independent experiments with 100 conidia examined in each replicate. Bar=10 µm. D) Statistical analysis of the appressorium melanin thickness (*n*=3 experiments, appressoria=15–30). Asterisks indicate significant differences (0.01, two‐tailed Student's *t*‐test). E) Melanin layers in appressorium of Guy11 and Δ*Moswi6* were examined under TEM (Hitachi HT‐7800 80KV). Red arrows indicate melanin layers. F) qRT‐PCR analyses the expression level of three melanin‐biosynthesis genes. Error bars are standard deviations from three biological replicates, and asterisks indicate significant differences (*p* < 0.01, two‐tailed Student's *t*‐test). G) Appressorium turgor was determined by 1.0–4.0 m concentration of glycerol solution treatment. Error bars are standard deviations from three biological replicates, with 100 appressoria counted per replicate. Asterisks represent significant differences (*p* < 0.01, two‐tailed Student's *t*‐test).

**Figure 5:**
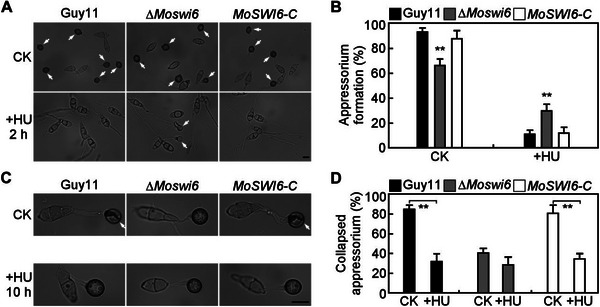
MoSwi6 plays a crucial role in turgor‐dependent S‐phase progression during appressorium maturation. A) Appressorium formation of the indicated strains exposure to 200 mm HU, added at 2 hpi and observed at 24 hpi. White arrows indicate appressoria. CK, ddH_2_O control. Bar = 10 µm. B) Statistical analysis of the appressorium formation with or without HU treatment. Error bars are standard deviations from three biological replicates with 100 conidia counted per replicate, and asterisks represent significant differences (*p* < 0.01, two‐tailed Student's *t*‐test). C) Effect of 4 m glycerol to appressoria with or without 200 mm HU treatment at 10 hpi, and observed at 24 hpi. White arrows indicate collapsed appressoria. Bar = 10 µm. D) Statistical analysis of the collapsed appressorium with or without HU treatment. Error bars are standard deviations from three biological replicates with 100 appressoria counted per replicate, and asterisks represent significant differences (*p* < 0.01, two‐tailed Student's *t*‐test).

To further characterize the underlying mechanism, we used MoMtg1 as a bait to screen the yeast cDNA library of *M. oryzae* and identified MoSwi6 that physically interacted with MoMtg1 by yeast two‐hybrid and co‐immunoprecipitation (co‐IP) assays (**Figure** **4**A,B). MoSwi6 is a transcription factor critical for appressorium‐mediated penetration.^[^ ^24^ ^]^ Appressorium‐related phenotype analysis revealed that the ∆*Moswi6* mutant exhibits similar defects to ∆*Momtg1*, with a thicker melanin layer, higher expression level of melanin biosynthesis genes, and an increased turgor pressure (Figure 4C–G), as well as abnormal ROS production and septin assembly (Figure S4A–D, Supporting Information). We further investigate the role of MoSwi6 in cell cycle during appressorium formation by treatments with HU. The results showed that, like the Δ*Momtg1* mutant, the Δ*Moswi6* mutant also formed appressoria in the presence of HU at 2 hpi and had similar higher appressorium turgor pressure with or without HU treatment at 10 hpi (**Figure** **5**A–D), suggesting that MoSwi6 is also essential for successful S‐phase transition during appressorium morphogenesis.

### 2.6 MoMtg1 Inhibits the Transcription Activity of MoSwi6 by Targeting *MoCYC1*

**Figure 6:**
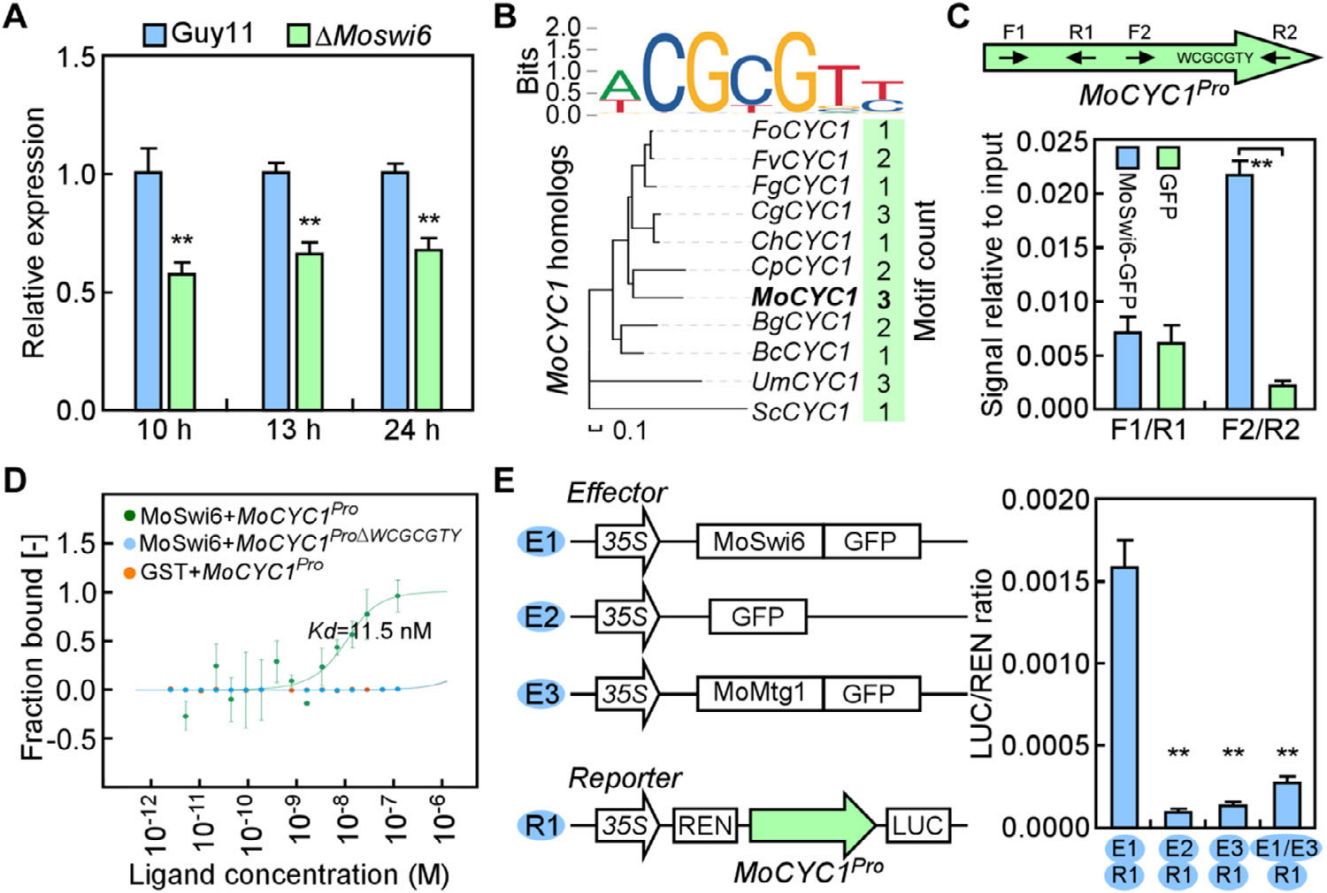
MoMtg1 inhibits the transcription activity of MoSwi6 that targeting the cyclin gene *MoCYC1*. A) Assays of *MoCYC1* expression during appressorium in the wild type Guy11 and ∆*Moswi6* mutant. Error bars are standard deviations from three biological replicates, and asterisks indicate significant differences (*p* < 0.01, two‐tailed Student's *t*‐test). B) The conserved MoSwi6‐binding site in *MoCYC1* and its orthologs from other fungi, including *Fusarium oxysporum*, *Fusarium verticillioides*, *Fusarium graminearum*, *Colletotrichum graminicola*, *Colletotrichum higginsianum*, *Claviceps purpurea*, *Magnaporthe oryzae*, *Blumeria graminis*, *Botrytis cinerea*, *Ustilago maydis* and *Saccharomyces cerevisiae*. C) ChIP‐qPCR analyses with the binding of MoSwi6 to the promoter region of *MoCYC1* (*MoCYC1^Pro^*). F1/R1 and F2/R2 indicate the primers to amplify DNA fragments. Error bars are standard deviations from three biological replicates, and asterisks indicate significant differences (*p* < 0.01, two‐tailed Student's *t*‐test). D) MST assays for the binding of MoSwi6 to the *MoCYC1* promoter with or without the predicted motif. (E) Luciferase assays of the transcriptional activity of MoSwi6 with or without the addition of MoMtg1 in vitro. Error bars are standard deviations from three biological replicates, and asterisks indicate significant differences (*p* < 0.01, two‐tailed Student's *t*‐test).

The B‐type cyclin gene *MoCYC1* is involved in cell cycle progression and in response to DNA damages.^[^ ^17^, ^25^ ^]^ Therefore, we analyzed the expression of *MoCYC1* in the wild type Guy11 and ∆*Moswi6* mutant, and found that the expression level of *MoCYC1* was significantly decreased in the mutant (**Figure** **6**A). To determine whether *MoCYC1* is the direct downstream target of MoSwi6, we conducted chromatin immunoprecipitation (ChIP)‐qPCR and microscale thermophoresis (MST) assays and showed that MoSwi6 bound to the promoter region of *MoCYC1* via the W(A/T)CGCGTY(G/C) motif (Figure 6C,D), which is conserved across fungi (Figure 6B). These results indicate that MoSwi6 is involved in cell cycle by directly regulating the expression of *MoCYC1*. We further investigated the biological meanings of the interaction between MoMtg1 and MoSwi6 and measured the transcriptional activity of MoSwi6 with the luciferase reporter under the control of the *MoCYC1* promoter in the presence of MoMtg1 or not. The results showed that MoMtg1 decreased the transcriptional activity of MoSwi6. Compared to the luciferase activity with MoSwi6 alone, the luciferase activity was significantly decreased when co‐incubated with MoMtg1 and MoSwi6. In treatments with MoMtg1 alone or green fluorescent protein (GFP) control, very low luciferase activity was detected (Figure 6E). These results demonstrate that MoMtg1 inhibits the transcription activity of MoSwi6 that targets the cyclin gene *MoCYC1*, thus controlling the turgor‐dependent S‐phase progression.

### 2.7 The Proper Expression Level of *MoCYC1* is Critical to Maintain Cell Cycle‐Mediated Appressorium Penetration

**Figure 7:**
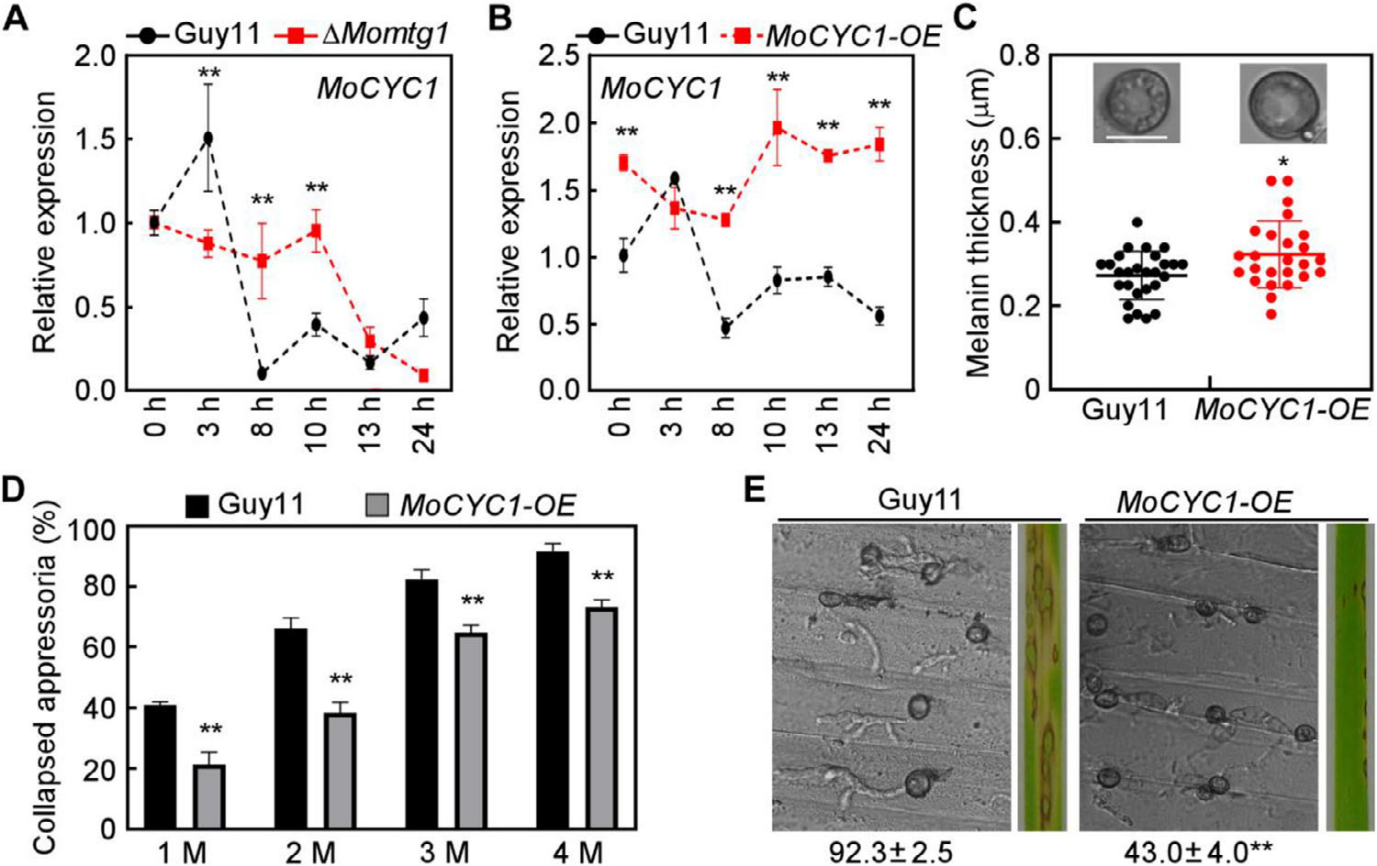
The proper expression level of *MoCYC1* is critical to maintain cell cycle‐mediated appressorium penetration. A) qRT‐PCR analyses the expression level of *MoCYC1* at different developmental stages of appressorium in the wild type Guy11 and ∆*Momtg1* mutant. Error bars are standard deviations from three biological replicates. B) qRT‐PCR analyses the expression level of *MoCYC1* in Guy11 and *MoCYC1‐OE* transformant. Error bars are standard deviations from three biological replicates. C) Appressorium melanin observation and thickness analysis of Guy11 and *MoCYC1‐OE* (*n* = 3 experiments, appressoria = 25–27). Bar = 10 µm. D) Statistical analyses the collapsed appressoria under 1–4 m glycerol treatment. Error bars are standard deviations from three biological replicates with 100 appressoria counted per replicate. E) Appressorium penetration efficiency of Guy11 and *MoCYC1‐OE* transformant on barley leaves at 24 hpi, and pathogenicity on rice seedlings at 7 dpi. The number indicates the percentage of penetration rate. Data from three independent experiments are shown as means ± SD (each experiment quantified 100 infection sites). All the above asterisks represent significant differences at *p* < 0.01(**) or *p* < 0.05(*), two‐tailed Student's *t*‐test.

Because similar defects of ∆*Momtg1* and ∆*Moswi6* in cell cycle‐mediated appressorium penetration and reduced expression of *MoCYC1* in ∆*Moswi6*, we then assayed the expression of *MoCYC1* in ∆*Momtg1* during appressorium development. In comparison with the wild type, the expression level of *MoCYC1* in ∆*Momtg1* was significantly higher during turgor‐dependent G1/S‐phase (8–10 hpi) (**Figure** **7**A). Although the ∆*Momtg1* and ∆*Moswi6* mutants had similar defects but deletion of these two genes had the opposite effects on *MoCYC1* expression, we hypothesized that either too low or too high level of *MoCYC1* expression will disrupt the normal turgor‐dependent G1/S‐phase transition. To test the possibility, we overexpressed *MoCYC1* in the wild type Guy11 and obtained the *MoCYC1‐OE* transformants (Figure 7B). As cyclin B accumulation is controlled transcriptionally,^[^ ^26^ ^]^ we found that *MoCYC1* overexpression significantly elevated the abundance of MoCyc1 proteins during appressorium maturation (Figure S5, Supporting Information). Phenotypic analysis revealed that *MoCYC1‐OE* exhibited hyper‐melanized appressoria with increased turgor, as well as reduced penetration efficiency and pathogenicity (Figure 7C–E). These results suggest that the proper regulation of *MoCYC1* expression during appressorium differentiation is critical to maintain normal cell cycle‐mediated appressoirum development and penetration.

### 2.8 Mtg1 is a Conserved Virulence Factor Unique to Filamentous Fungi

**Figure 8:**
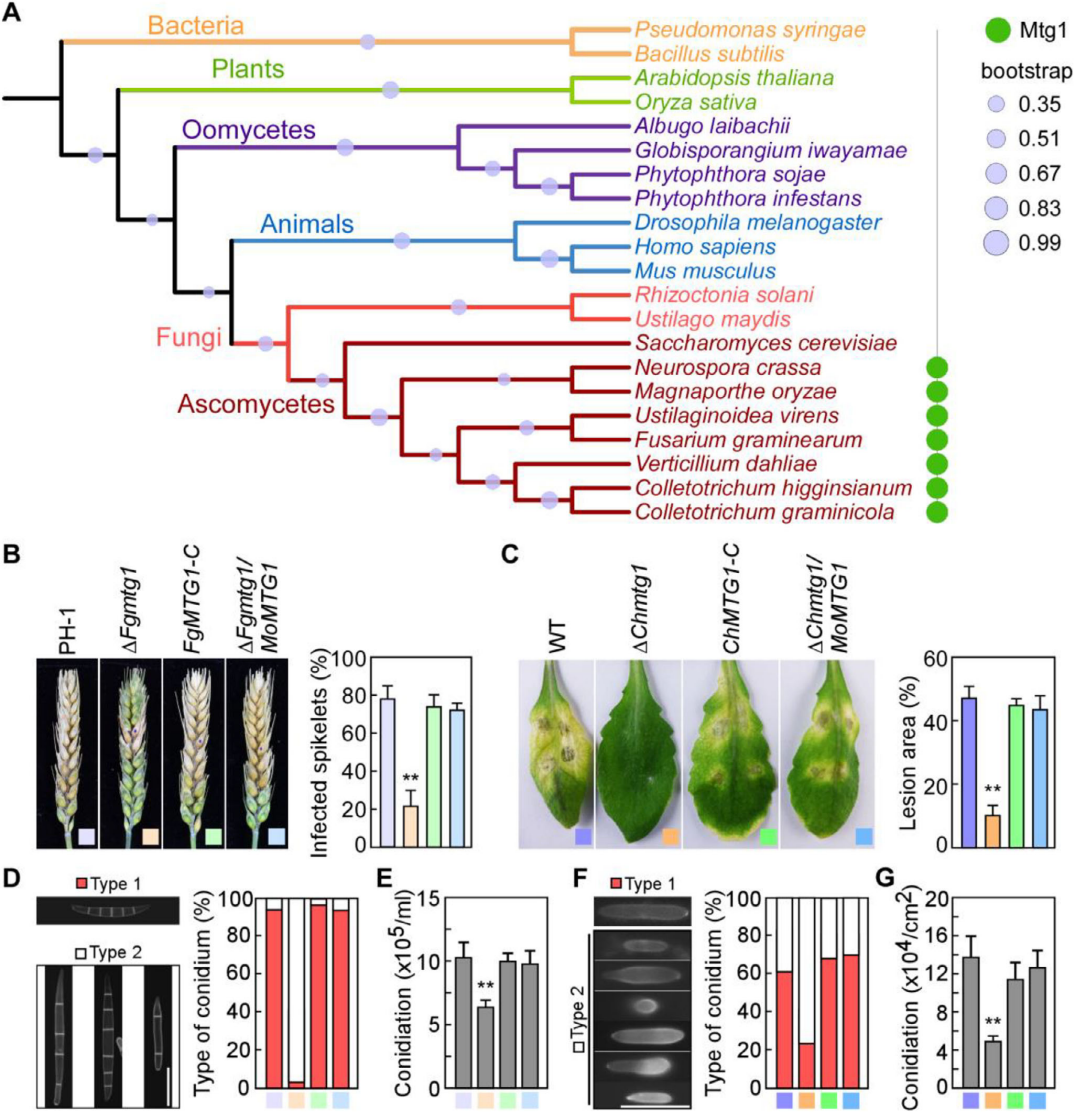
Mtg1 only exists in filamentous ascomycetes and is conserved in both sequence and function. A) Phylogenetic distribution of MoMtg1 across orthologs representative lineages of fungi, oomycetes, plants, animals, and bacteria. B) Pathogenicity assay of the Δ*Fgmtg1* mutant on wheat and the percentage of infected spikelets was statistically analyzed across three independent biological replicates. For each replicate, 10 wheat heads were inoculated and scored. Asterisks indicate significant differences (*p* < 0.01, two‐tailed Student's *t*‐test). C) Pathogenicity assay of the Δ*Chmtg1* mutant on *Arabidopsis thaliana* and the lesion area was statistically analyzed. For three independent biological replicates, 3 inoculated leaves per replicate were measured using ImageJ. Asterisks indicate significant differences (*p* < 0.01, two‐tailed Student's *t*‐test). D and F) Statistical analyses the conidial morphology of the indicated strains. Bar = 10 µm. (E and G) Statistical analyses the conidial production of the indicated strains. Error bars are standard deviations from three biological replicates, and asterisks represent significant differences (*p* < 0.01, two‐tailed Student's *t*‐test).

Phylogenetic analysis indicates that MoMtg1 and its homologs are conserved among filamentous fungi. However, they are not found in other organisms, including *Homo sapiens*, other animals, plants and oomycetes (**Figure** **8**A; Figure S6, Supporting Information). To test whether the biological role of Mtg1 proteins are conserved in pathogenic fungi, we identified the MoMtg1 homologs in the wheat head scab fungus *Fusarium graminearum* and anthracnose pathogen *Colletotrichum higginsianum* and generated mutants deleted of *FgMTG1* and *ChMTG1* (Figure S7A–D, Supporting Information), that share 41.5% and 41.2% amino acid identity with MoMtg1. Similar to Δ*Momtg1*, the virulence of Δ*Fgmtg1* and Δ*Chmtg1* mutants is significantly reduced compared to that of the wild type and complemented transformant (Figure 8B,C). Additionally, conidiation and conidium morphology were also defective in these two mutants (Figure 8D–G). Moreover, heterogenous expression of *MoMTG1* in Δ*Fgmtg1* and Δ*Chmtg1* was able to rescue their defects in conidiogenesis and pathogenicity (Figure 8B–G). These findings indicate that MoMtg1 and its homologs are conserved and serve as critical proteins in governing the development and pathogenicity of various fungal pathogens.

### 2.9 HIGS of MoMtg1 Enhances Resistance to Rice Blast in Rice without Agronomic Trait Defects

**Figure 9:**
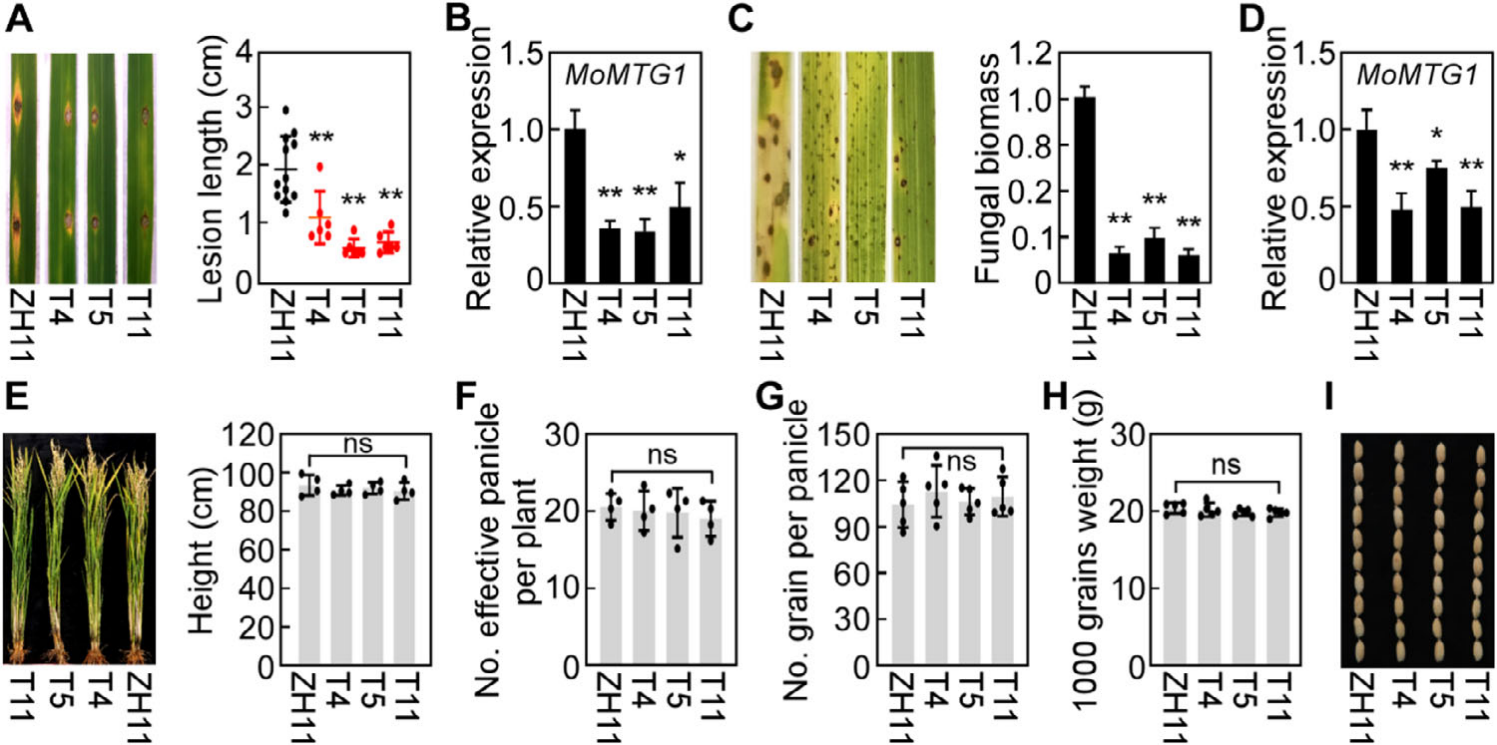
HIGS of MoMtg1 enhances resistance to rice blast in rice without agronomic trait defects. A) Punch assay analyses rice blast resistance in T4, T5, and T11 transgenic rice plants, and the lesion length was statistically analyzed, each using 3‐6 leaves from different plants per transgenic line. B and D) The expression level of *MoMTG1* in transgenic rice plants assayed by qRT‐PCR. Error bars are standard deviations from three biological replicates. C) Representative leaves of T4, T5, and T11 transgenic rice plants sprayed with Guy11 (left) and assayed for fungal biomass by qRT‐PCR. Error bars are standard deviations from three biological replicates. E–I) The T4, T5, and T11 transgenic rice plants were assayed for the height E), panicle number per plant F), grain number per panicle G), the weight of 1000 grains H), and grain size I). Mean and standard deviations were estimated with data from three biological replicates, with at least four samples examined in each replicate. All the above asterisks represent significant differences at *p* < 0.01(**) or *p*<0.05(*), two‐tailed Student's *t*‐test; ns, no significance.

The host‐induced gene silencing (HIGS) approach based on RNA interference (RNAi) has been broadly used in creating new disease‐resistant crop varieties against fungal diseases.^[^ ^27^ ^]^ Based on the conservation of Mtg1 in protein sequences and their biological functions, we used MoMtg1 as the target to generate transgenic rice plant by expressing *MoMTG1* siRNA in rice susceptible cultivar ZH11. Three positive transgenic rice plants (T4, T5, and T11) were identified and phenotypically analyzed. In comparison to ZH11, transgenic plants T4, T5, and T11 were increased in resistance to *M. oryzae* in both punch and spraying assays. Meanwhile, the expression level of *MoMTG1* was significantly decreased in *M. oryzae*‐infected tissues of T4, T5 and T11 plants (**Figure** **9**A–F). These results suggest that expression of this RNAi construct in rice lead to the silence of *MoMTG1* in *M. oryzae* upon infection, making these transgenic rice lines as blast‐resistance cultivars. Because high‐levels of disease resistance often lead to penalty in crop yield,^[^ ^28^ ^]^ T4, T5, and T11 plants were assayed for plant height, panicle number, grain number per panicle, thousand‐grain weight, panicle morphology, and grain size. We found that none of these agronomic traits was remarkably altered in all three transgenic plants compared to ZH11 (Figure 9G–I). These results indicate that T4, T5, and T11 generated by HIGS have increased disease resistance but no penalty in crop yield and other defects in agronomic traits.

### 2.10 Compound ZS1619 Blocks Appressorium‐Mediated Penetration by Impairing MoMtg1 Function

**Figure 10:**
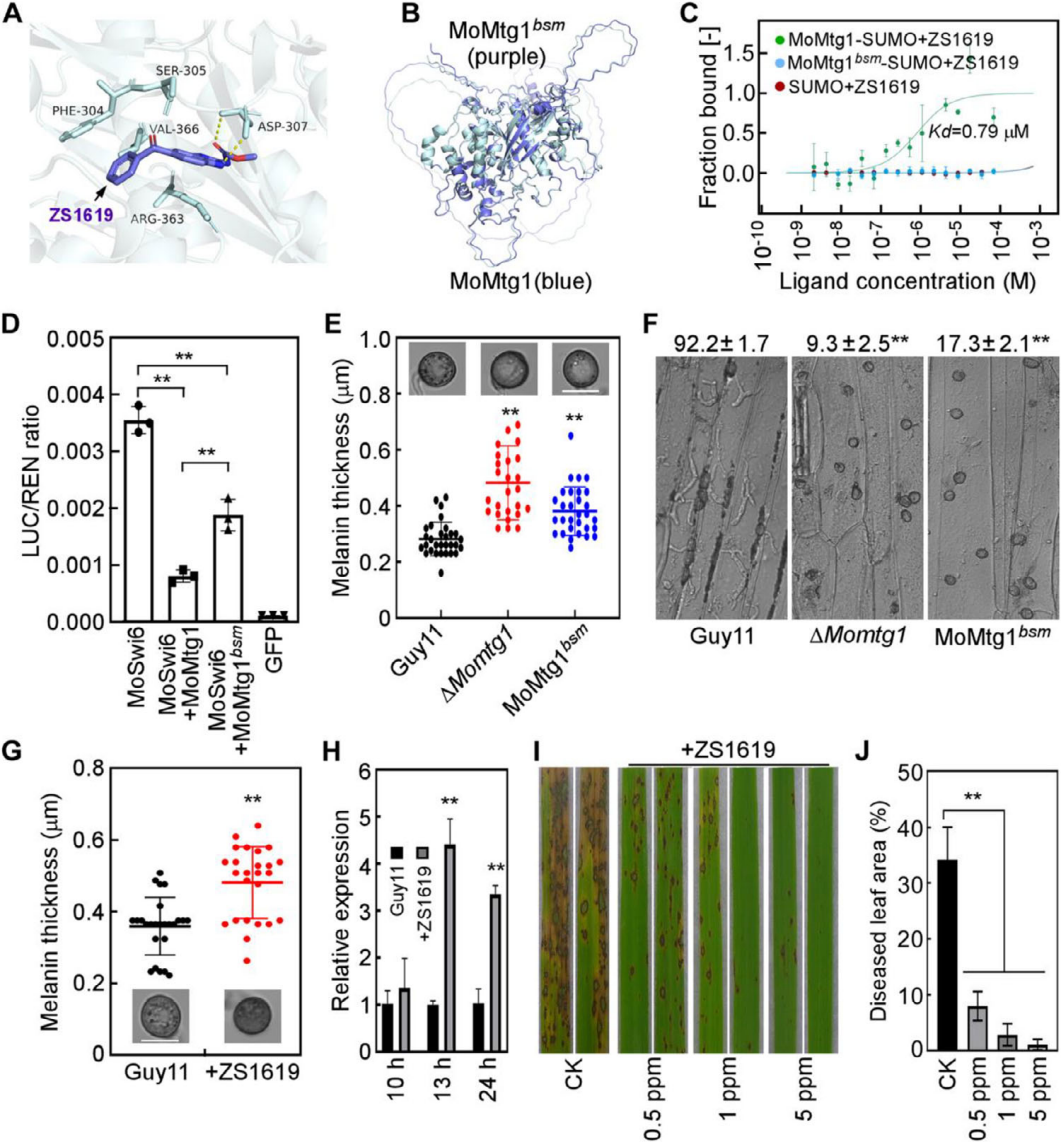
Compound ZS1619 blocks appressorium‐mediated penetration by impairing MoMtg1 function. A) Molecular docking between MoMtg1 and ZS1619. B) Homology modeling of MoMtg1 and Mutation sites in MoMtg1*^bsm^*. C) MST assays of the affinity between ZS1619 and MoMtg1 or MoMtg1*^bsm^*. D) Luciferase assays of MoSwi6 transcription activity with the addition of MoMtg1 or MoMtg1*^bsm^* proteins in vitro. Error bars are standard deviations from three biological replicates, and asterisks indicate significant differences (*p* < 0.01, two‐tailed Student's *t*‐test). E) Appressorium melanin observation and thickness analysis Guy11, ∆*Momtg1* and MoMtg1*^bsm^* (*n* = 3 experiments, appressoria = 26–30). F) Appressorium penetration efficiency of Guy11, ∆*Momtg1* and MoMtg1*^bsm^* on barley leaves at 24 hpi. The number indicates the percentage of penetration rate. Mean and SD are calculated with data from three independent experiments, with 100 infection sites examined in each experiment. Asterisks represent significant differences (*p* < 0.01, two‐tailed Student's *t*‐test). G) Appressorium melanin observation and thickness analysis of the wild type Guy11 with or without 1 ppm ZS1619 treatment (*n* = 3 experiments, appressoria = 25). H) qRT‐PCR analyses the expression level of *MoCYC1* in Guy11 with or without 1 ppm ZS1619 treatment. Error bars are standard deviations from three biological replicates, and asterisks represent significant differences (*p* < 0.01, two‐tailed Student's *t*‐test). I) Conidial suspensions of Guy11 were sprayed onto rice seedlings with or without different concentrations of ZS1619, and photographed at 6 dpi. J) The diseased leaf area was statistically analyzed using ImageJ. Error bars represent standard deviations from three biological replicates (8 leaves measured per replicate), and asterisks represent significant differences (*p* < 0.01, two‐tailed Student's *t*‐test).

Developing targeted fungicides is a popular and crucial research area in plant protection. However, the scarcity of available targets severely impedes the development of targeted fungicide.^[^ ^29^ ^]^ To evaluate whether MoMtg1 could serve as a viable fungicide target, we first predicted its three‐dimensional (3D) structure using AlphaFold3. Subsequently, we screened small molecule compounds that could bind to MoMtg1 via virtual molecular docking. A total of 10 compounds with high affinity scores were identified. Among them, ZS1619 exhibited the most significant effect on appressorium turgor, with five predicted binding sites (F304, S305, D307, R363, V366) (**Figure** **10**A). We then mutated these five sites to create MoMtg1*^bsm^* and found that its 3D structure is very similar to that of MoMtg1, with an root mean square deviation (RMSD) score of 0.332Å in the alignment between the two structures (Figure 10B). However, the binding of ZS1619 to MoMtg1*^bsm^* was disrupted (Figure 10C). A luciferase assay further revealed a significant decrease in the ability of MoMtg1*^bsm^* to repress the transcriptional activity of MoSwi6 (Figure 10D). Phenotypic analysis revealed that transformants expressing MoMtg1*^bsm^* formed hyper‐melanized appressoria, a similar phenomenon observed in Δ*Momtg1* (Figure 10E). Moreover, the penetration efficiency on barley leaves was remarkably decreased in the MoMtg1*^bsm^* transformant (Figure 10F). These results indicated that these five sites targeted by ZS1619 are critical for the full function of MoMtg1. We then accessed the effect of ZS1619 on appressorium‐mediated infection processes, and found that treatments with ZS1619 resulted in the formation of hyper‐melanized appressoria and high expression levels of *MoCYC1* in the wild type (Figure 10G,H), which is similar to that found in Δ*Momtg1*. Furthermore, spraying assays showed that the pathogenicity of Guy11 was significantly reduced on rice seedlings treated with ZS1619 (Figure 10I,J). Taken together, these findings indicated that MoMtg1 is a promising fungicide target that can be targeted by ZS1619 to control the rice blast disease.

## 3 Discussion

**Figure 11:**
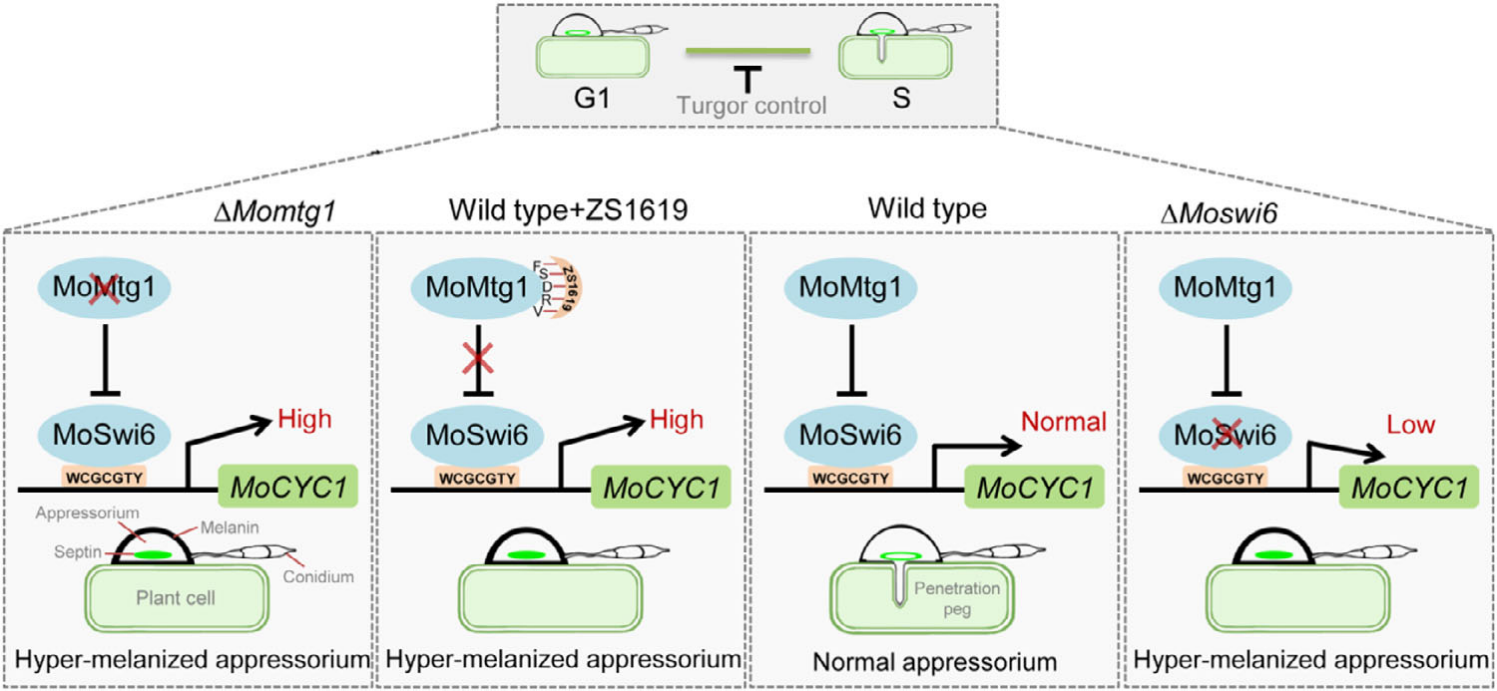
The working model of MoMtg1 in appressorium mediated penetration. During the turgor‐mediated G1/S phase progression in the appressorium, MoSwi6 plays a role in cell cycle regulation by activating *MoCYC1* transcription. MoMtg1, acting as a transcriptional repressor, works with MoSwi6 to ensure *MoCYC1* is expressed at a moderate level, which is vital for the appressorium's cell cycle‐related functions. Compound ZS1619 can bind to MoMtg1 specifically to inhibit its function, thus impacting the appressorium‐mediated penetration.

Appressorium formation and plant penetration is intricately connected to cell cycle progression in *M. oryzae*.^[^ ^18^ ^]^ Previous studies have shown that the S‐phase progression is essential for appressorium maturation, repolarization and penetration.^[^ ^17^ ^]^ In this study, we identified a novel protein, MoMtg1, that functions as the transcriptional repressor of MoSwi6 during turgor‐dependent cell cycle progression to regulate the expression of *MoCYC1*, ensuring appressorium‐mediated penetration. Furthermore, a small molecule compound, ZS1619, was identified as an inhibitor of MoMtg1 that is effective for controlling the rice blast disease (**Figure** **11**). Together with results from HIGS silencing of *MoMTG1*, we demonstrated that MoMtg1 is a promising new target for novel fungicide design and disease control.

During appressorium maturation, the accumulation of a certain level of turgor is a necessary condition for the S‐phase progression. Once the turgor reached a maximum, repolarization occurs with the formation of a penetration peg.^[^ ^13^ ^]^ Appressorium turgor generation requires melanin synthesis. Excessive turgor caused by S‐phase arrest leads to an abnormal thick melanin layer, resulting in defects in the reestablishment of polarized growth, penetration peg formation, and plant penetration.^[^ ^17^, ^30^ ^]^ We found that Δ*Momtg1* and Δ*Moswi6* appressoria had similar defects in S‐phase arrest, indicating their functional relationship with cell cycle control. Treatments with HU at 10 h had no obvious effects on the generation of appressorium turgor in the Δ*Momtg1* and Δ*Moswi6* mutants. In addition, the S‐phase arrest at 2 h was bypassed by HU in mutants deleted of *MoMTG1* and *MoSWI6*. These observations indicate that MoMtg1 and MoSwi6 are involved in regulating the progression through the S phase in the cell cycle during appressorium formation in *M. oryzae*.

Upon activation of the turgor‐dependent S‐phase checkpoint, the Nox2/NoxR complex mediates ROS accumulation that controls septin recruitment and cytoskeletal remodeling.^[^ ^23^ ^]^ Our results indicated that deletion of MoMtg1 and MoSwi6 did not impair the generation of ROS but resulted in a defect in the degradation of intracellular ROS during appressorium maturation. Previous studies have demonstrated that excessive accumulation of ROS within plant cells can cause DNA damages and plant developments.^[^ ^31^, ^32^ ^]^ It is likely that abnormal accumulation of ROS is also a significant cause of impaired appressorium function in *M. oryzae*.

As the common activating subunit of the SCB‐binding factor (SBF) and MCB‐binding factor (MBF) transcription factor complexes in the budding yeast, Swi6 plays a critical role in the cell cycle and regulates the expression of a number of cell cycle‐related genes, including those in the cyclin family.^[^ ^33^, ^34^, ^35^ ^]^ During G1 phase, the activated SBF triggers high expression level of the G1 cyclin genes *CLN1* and *CLN2*, driving the transition from G1 to S phase.^[^ ^36^, ^37^ ^]^ The Swi6 homolog in *M. oryzae*, MoSwi6, has been identified as a downstream effector of MoMps1, participating in cell wall biosynthesis and appressorium‐mediated penetration.^[^ ^24^ ^]^ Our research demonstrated its functions in the cell cycle progression. Interestingly, unlike Swi6, an activating subunit in *S. cerevisiae*, MoSwi6 has a DNA binding domain. We showed that MoSwi6 could bind to the promoter of the B‐type cyclin gene *MoCYC1* and exhibited transcriptional activation activity. Therefore, MoSwi6 may function as an independent transcription factor in regulating cell cycle progression in *M. oryzae*. Cyc1 is a component of the B‐type cyclin‐Cdk1 complex, which is known to be required for the S‐phase during appressorium morphogenesis and plant infection in the rice blast fungus.^[^ ^17^ ^]^ MoSwi6 may play a role in the progression of the S‐phase by activating the transcription of *MoCYC1*, thereby participating in appressorium‐mediated penetration.

In eukaryotic cells, the cyclin B‐Cdk1 complex is essential for cell cycle progression, particularly in mediating the transition from the S phase to the G2 phase. Its activation is contingent upon the transcriptional levels of cyclins.^[^ ^38^, ^39^ ^]^ However, the precise regulation of cyclin B expression is critical. Previous studies have indicated that excessive expression of cyclin B can shorten the S phase, which leads to entry into the G2 phase before the completion of DNA replication, resulting in cell cycle progression disorders.^[^ ^20^ ^]^ Our data reveal that the transcriptional pattern of *MoCYC1* during appressorium formation and plant infection exhibits cyclical fluctuations, with significant upregulation at the initiation, followed by a decline as the cells progressing through the cell cycle. In contrast, the *MoCYC1* transcription level in the Δ*Momtg1* mutant during the S phase was significantly higher than in the wild‐type. Furthermore, MoMtg1 inhibits the transcription activity of MoSwi6 that activates the transcription of *MoCYC1*. These findings imply a potential role for the interaction between MoMtg1 and MoSwi6 in controlling *MoCYC1* expression within a normal threshold. The coordinated control of *MoCYC1* transcription is crucial for the cell cycle‐regulated appressorium maturation, because overexpression of *MoCYC1* leads to excessive MoCyc1 accumulation that impairs normal cell cycle progression during appressorium development and turgor‐dependent penetration.

In agricultural practice, the prolonged monoculture of single‐gene‐resistant cultivars coupled and repeated applications of fungicides with similar modes of action has driven the rapid evolution of resistance in fungal pathogen populations.^[^ ^40^, ^41^ ^]^ Given this situation, creating disease‐resistance cultivars by unconventional approach such as HIGS and exploring new targets for novel fungicide development are necessary and have great potential for controlling crop diseases.^[^ ^27^, ^42^ ^]^ As a conserved protein unique to filamentous fungi and a critical regulator in appressorium penetration, MoMtg1 is a candidate target with potential dual applications to effectively control rice blast. Our experiments show that the HIGS transgenic rice targeting *MoMTG1* for silencing displayed enhanced resistance against *M. oryzae* without negative impacts on agronomic traits. Moreover, through AI‐based virtual screening, we discovered that ZS1619 is an inhibitor of MoMtg1 and holds significant potential in fungicide development. Notably, in contrast to traditional targets such as the dihydroxynaphthalene (DHN)‐melanin biosynthesis inhibited by tricyclazole with a limited application to control pathogens like *M. oryzae* relying on melanin for infection or survival,^[^ ^43^, ^44^ ^]^ MoMtg1 acts as a multi‐pathway regulator that coordinates, both in cell cycle progression and ROS homeostasis during appressorium development. The multiple functions and broad phylogenetic conservation positions it as a potential broad‐spectrum target. In addition, application of HIGS‐*MoMTG1i* resistant lines combined with MoMtg1‐targeting fungicide may establish a dual‐selection barrier, which significantly raises the genetic barrier for *M. oryzae* to develop resistance mutations capable of overcoming both targets, thereby reducing risks of resistance development associated with single control tactics.

## 4 Experimental Section

### Fungal Strains, Culture Conditions, and Appressorium Development Assays

The *M. oryzae* isolate Guy11, the *C. higginsianum* isolate IMI, and the *F. graminearum* isolate PH‐1 were used as wild type (WT) strains in this study. For *M. oryzae* vegetative growth, 3 mm×3 mm agar blocks were placed on complete medium (CM) and incubated at 28 °C in the dark for 7 days and colony diameters were subsequently measured.^[^ ^45^ ^]^ For conidiation, mycelial blocks of *M. oryzae* were incubated on rice and corn media (SDC) for 5 days, following illumination with black light for 3 days;^[^ ^46^ ^]^ mycelia of *C. higginsianum* were grown on potato dextrose agar plate at 25 °C at 12 h under black‐light blue fluorescent bulb light/12 h dark for 10 days,^[^ ^47^ ^]^ and mycelial blocks of *F. graminearum* were inoculated in 100 ml carboxymethylcellulose (CMC) liquid medium and incubated at 25 °C, 150 rpm for 5 days.^[^ ^48^ ^]^

### Target Gene Deletion and Complementation

To generate gene deletion mutants, 1.0 kb sequences from upstream and downstream fragments of the target genes were amplified from the genomic DNA of the WT strain Guy11, and then linked to the hygromycin phosphotransferase cassette released from pCX62. The resulting fragments were transformed into protoplasts of the wild‐type strains for mutation screening.^[^ ^49^ ^]^ To generate the complemented transformants, the full‐length *MTG1* (excluding the terminator) along with its native promoter was amplified and cloned into the pYF11 vector by the yeast gap‐repair approach.^[^ ^50^ ^]^ The recombined constructs were introduced into the corresponding mutants and screened by bleomycin resistance and GFP signal.

### Pathogenicity Assays

Conidia from the tested strains were washed by ddH_2_O and filtered through one layer of Miracloth (EMD Millipore Corp., 475855‐1R). For *M. oryzae*, the conidial suspensions were adjusted to a concentration of 1×10^5^ spores/ml in 0.2% (w/v) gelatin solution. Pathogenicity assays were performed as previously described.^[^ ^51^ ^]^ Diseased leaves were photographed at 7 days (rice) or 5 days (barley) post‐inoculation (dpi). For transgenic rice resistance test, 40‐day‐old rice plants were used for punch inoculation. Conidial suspensions (5×10^5^ spores mL^−1^) were inoculated onto the injured area of the leaves wounded with a hole punch and measured the lesion area and fungal biomass at 7 dpi. For *C. higginsianum*, 5‐week‐old *Arabidopsis thaliana* seedlings were inoculated with mycelial blocks and kept in a growth chamber at 22 °C under an 8 h light/16 h dark cycle. The inoculated leaves were photographed at 7 dpi.^[^ ^52^ ^]^ The degree of disease lesions was analyzed by ImageJ.^[^ ^49^ ^]^ For *F. graminearum*, 6‐week‐old wheat cultivar with flowering heads was used for infection assays. The conidial suspensions (1×10^6^ spores mL^−1^) were injected into the spikelet of the flowering wheat head, and each head inoculated 10 µl conidial suspensions. Infected spikelets in each head were counted and analyzed at 14 dpi.^[^ ^53^ ^]^

To examine the *M. oryzae* penetration and invasive growth, three‐week‐old rice sheaths were chosen for injection with conidial suspensions (3×10^5^ spores mL^−1^) and incubated at 28 °C in the dark. After 24 h the inner epidermal cells of above sheaths were inspected under a microscope.^[^ ^54^ ^]^

### Appressorium Formation, Melanin Thickness Quantification, Turgor Measurement and ROS Detection Assays

For appressorium formation, conidial suspensions (1×10^5^ spores mL^−1^) were inoculated onto hydrophobic glass cover slips (Epredia) and incubated at 28 °C in the dark. For melanin thickness quantification, appressoria at 24 hpi were imaged using a Zeiss LSM980 confocal microscope. The melanin layer thickness was measured using the Profile module in ZEN 3.3 software by analyzing intensity profiles over line scan diameters crossing the appressorium center, as previously described.^[^ ^17^ ^]^ The turgor pressure was measured by cell collapse assays at 24 h as described previously,^[^ ^55^ ^]^ and observed by Zeiss Axio Observer A1 inverted microscope. Molecular probes DCFH‐DA was used to detect intracellular ROS level. Appressorium at different time points were incubated in 10 µM DCFH‐DA (Beyotime, S0033S) for 20 min at 37 °C in the dark and washed with ddH_2_O for three times and observed by microscope under the green fluorescence channel.

### TEM Observation

Conidia of the tested strains were incubated on onion epidermis at 28 °C in the dark. After 24 h, epidermal sections with adhering appressoria were fixed in 2.5% glutaraldehyde and 2% paraformaldehyde for >4 h. Fixed materials were sent to the Institute of Plant Virology at Ningbo University for TEM analysis as previously described.^[^ ^51^ ^]^

### Subcellular Localization Assay

The full‐length of target genes (excluding the terminator) along with their native promoters was amplified from the WT strain Guy11 genomic DNA and cloned into the pYF11 vector by yeast gap‐repair approach.^[^ ^50^ ^]^ The resulting constructs pYF11‐*MoSEP4*‐GFP, pYF11‐*MoSEP5*‐GFP, pYF11‐*MoSWI6*‐GFP, pYF11‐H1‐RFP were transformed into Guy11 and Δ*Momtg1* mutant. The resulting transformants were observed under a confocal laser scanning microscope (ZEISS LSM980 with Airyscan 2, 63× oil) during appressorium development.

### Yeast‐Two‐Hybrid Assay

The cDNAs of target genes were cloned into the pGADT7 and pGBKT7 vectors. The resulting constructs were co‐transformed into the yeast strain AH109 using the protocol described previously.^[^ ^56^ ^]^ The positive transformants were selected on synthetic dextrose medium lacking tryptophan and leucine (SD‐Leu‐Trp), and further confirmed on medium lacking tryptophan, leucine, and histidine (SD‐His‐Leu‐Trp) with an appropriate concentration of 3‐aminotriazole (3‐AT).

### Co‐Immunoprecipitation (co‐IP) Assay

The recombination constructs MoMtg1‐GFP and MoSwi6‐flag were co‐transformed into wild type strain Guy11 and selected using hygromycin (HPH) and bleomycin (Ble) resistance. Total proteins that extracted from positive transformant were co‐incubated with anti‐GFP agarose beads (KT HEALTH, KTSM1301) for 4 h under cold, and then washed 3 times with 1× PBS. 200 mm glycine (pH 2.5) were used for eluting the protein bound to the beads, followed by 1 M Tris base (pH 10.4) as neutralization buffer. The total and eluted proteins were analyzed by Western blot.^[^ ^57^ ^]^

### RNA Isolation and Quantitative Real‐Time (qRT)‐PCR

Total RNA from appressoria inoculated on onion epidermis or rice leaves was isolated using the Omega Total RNA Kit I, following the manufacturer's instructions. For Quantitative RT‐PCR (qPCR), cDNA synthesis was synthesized using reverse transcriptase HiScript III RT SuperMix for qPCR (Vazyme Biotech Co., Nanjing, China). qRT‐PCR reactions were performed using the ChamQ Universal SYBR qPCR Master Mix (Q711, Vazyme, China). The 2^−∆∆CT^ method was used to calculate the transcription level for each sample, *M. oryzae ACTIN* gene serves as an internal reference control.^[^ ^58^ ^]^

### Chromatin Immuno‐Precipitation (ChIP) and ChIP‐qPCR Analysis

The ChIP protocol was carried out as previously described.^[^ ^59^ ^]^ Recombined transformants expressing the construct of MoSwi6‐GFP and GFP alone were utilized for chromatin isolation. Following sonication, the DNA/protein complex was immune‐precipitated with anti‐GFP agarose beads (KT HEALTH, KTSM1301). The immune‐precipitated DNA and input DNA were purified after reverse cross‐linking and protease K digestion. The above DNA samples were analyzed in qPCR, using primers that designed specific to MoSwi6 binding region in *MoCYC1* promoter. The results were calculated as the percentage of input DNA and comparing it to the signal from the GFP transformant: %input = 2% × 2^(Ct^Input^‐Ct^IP^). The ChIP‐qPCR assay was validated through three biological replicates.^[^ ^60^ ^]^

### Luciferase Activity Assays and MST Analysis

For Luciferase activity assays, the cDNA of *MoSWI6* and *MoMTG1* were inserted into the pBIN‐GFP vector as effector plasmids, the promoter fragment of *MoCYC1* and the putative binding motif mutation in *MoCYC1* promoter were cloned into pGreenII 0800‐LUC as reporter plasmids. The effector and reporter plasmids were co‐expressed in *Nicotiana benthamiana* cells via *Agrobacterium*‐mediated transformation. After incubation at 25 °C for 48 h, the transcriptional activity was determined using the Dual Luciferase Reporter Gene Assay Kit protocol (Beyotime, RG027).^[^ ^61^ ^]^ For MST analysis, binding reactions of *MoCYC1* promoter and its binding motif mutation fragment with recombinant protein GST‐Swi6 were measured by MST in a Monolith NT. LabelFree instrument (NanoTemper Technologies GmbH). The measurement was performed as described previously.^[^ ^46^ ^]^

### Phylogenetic Tree Construction

To construct the phylogenomic tree, the genomes of selected common species, including Pseudomonas syringae, Bacillus subtilis, Arabidopsis thaliana, Oryza sativa, Albugo laibachii, Globisporangium iwayamae, Phytophthora sojae, Phytophthora infestans, Drosophila melanogaster, Homo sapiens, Mus musculus, Rhizoctonia solani, Ustilago maydis, Saccharomyces cerevisiae, Neurospora crassa, Magnaporthe oryzae, Ustilaginoidea virens, Fusarium graminearum, Verticillium dahliae, Colletotrichum higginsianum and Colletotrichum graminicola, were downloaded from FungiDB and NCBI database, using orthofinder (version 3.0.1b1) to filter single‐copy orthologs present in all listed species.^[^ ^62^ ^]^ The phylogenomic tree was constructed using FastTree based on the alignments of these single‐copy ortholog families under the approximate maximum‐likelihood model with 1000 bootstrap replicates. To investigate the distribution of MoMtg1 orthologs across species, BLASTP searches were performed using the MoMtg1 sequence as a query against the NCBI non‐redundant (nr) protein database with an E‐value cut off of 1e‐3. Putative orthologous proteins identified were subsequently mapped onto the phylogenomic tree.

### Virtual Screening

According to the physical properties and molecular weight, the structure files of compounds were downloaded from the PubChem compound databases (https://pubchem.ncbi.nlm.nih.gov/). Convert SDF ligands to PDB format with explicit hydrogens using OpenBabel, and then prepare PDBQT files for docking via AutoDockTools with Gasteiger charges and rotatable bond definitions. Use AutoDock Vina to perform molecular docking. Sort according to the size of the affinity, and select appropriate compounds for next experiments. Use Python scripts to drive batch processing of the above processes.

### Statistical Analysis

Perform statistical analysis using GraphPad Prism 8.0.2 software. All experiments were performed at least three times. Statistical significance was calculated using two‐sided unpaired Student's *t*‐test as previously described.^[^ ^63^ ^]^ All error bars represent the standard deviation (SD) of the mean. Asterisks in all graphs indicate statistical significance (* *p* < 0.05; ** *p* < 0.01; ns, no significant).

## Conflict of Interest

The authors declare no conflict of interest.

## Author Contributions

X.W. and T.Z. contributed equally to this work. X.W., T.Z., Z.Z., and H.Z. designed the research; X.W., T.Z., Q.L., C.J., L.H., and X.L. performed the experiments; X.W., T.Z., Q.L., C.J., L.H., M.Z., X.L., M.L., and H.Z. analyzed the data; and X.W., L.Y., G.L., Z.Z., and H.Z. wrote the manuscript.

## Supporting information



Supporting Information

## Data Availability

The data that support the findings of this study are available from the corresponding author upon reasonable request.

## References

[1] N. B. Quoc , N. N. B. Chau , Curr. Protein Pept. Sci. 2017, 18, 1019. 27526928 10.2174/1389203717666160813164955

[2] N. J. Talbot , Curr. Biol:CB 2019, 29, R144. 30836078 10.1016/j.cub.2018.12.050

[3] N. J. Talbot , Annu. Rev. Microbiol. 2003, 57, 177. 14527276 10.1146/annurev.micro.57.030502.090957

[4] R. A. Wilson , N. J. Talbot , Nat. Rev. Microbiol. 2009, 7, 185. 19219052 10.1038/nrmicro2032

[5] X. Yan , N. J. Talbot , Curr. Opin. Microbiol. 2016, 34, 147. 27816794 10.1016/j.mib.2016.10.001

[6] J. Fernandez , K. Orth , Trends Microbiol. 2018, 26, 582. 29395728 10.1016/j.tim.2017.12.007 PMC6003838

[7] F. Nasir , L. Tian , C. Chang , X. Li , Y. Gao , L. P. Tran , C. Tian , Semin. Cell Dev. Biol. 2018, 83, 95. 29061483 10.1016/j.semcdb.2017.10.020

[8] B. N. Devanna , P. Jain , A. U. Solanke , A. Das , S. Thakur , P. K. Singh , M. Kumari , H. Dubey , R. Jaswal , D. Pawar , R. Kapoor , J. Singh , K. Arora , B. K. Saklani , C. AnilKumar , S. M. Maganti , H. Sonah , R. Deshmukh , R. Rathour , T. R. Sharma , J. Fungi (Basel) 2022, 8, 584. 35736067 10.3390/jof8060584 PMC9224618

[9] C. Veneault‐Fourrey , M. Barooah , M. Egan , G. Wakley , N. J. Talbot , Science 2006, 312, 580. 16645096 10.1126/science.1124550

[10] J. C. de Jong , B. J. McCormack , N. Smirnoff , N. J. Talbot , Nature 1997, 389, 244.

[11] Y. F. Dagdas , K. Yoshino , G. Dagdas , L. S. Ryder , E. Bielska , G. Steinberg , N. J. Talbot , Science 2012, 336, 1590. 22723425 10.1126/science.1222934

[12] M. J. Egan , Z. Y. Wang , M. A. Jones , N. Smirnoff , N. J. Talbot , Proc. Natl. Acad. Sci. USA 2007, 104, 11772. 17600089 10.1073/pnas.0700574104 PMC1913907

[13] L. S. Ryder , Y. F. Dagdas , M. J. Kershaw , C. Venkataraman , A. Madzvamuse , X. Yan , N. Cruz‐Mireles , D. M. Soanes , M. Oses‐Ruiz , V. Styles , J. Sklenar , F. L. H. Menke , N. J. Talbot , Nature 2019, 574, 423. 31597961 10.1038/s41586-019-1637-x

[14] N. Dulal , A. M. Rogers , R. Proko , B. D. Bieger , R. Liyanage , V. R. Krishnamurthi , Y. Wang , M. J. Egan , J. Cell Sci. 2021, 134, jcs251298. 33414165 10.1242/jcs.251298

[15] M. He , J. Su , Y. Xu , J. Chen , M. Chern , M. Lei , T. Qi , Z. Wang , L. S. Ryder , B. Tang , M. Osés‐Ruiz , K. Zhu , Y. Cao , X. Yan , I. Eisermann , Y. Luo , W. Li , J. Wang , J. Yin , S. M. Lam , G. Peng , X. Sun , X. Zhu , B. Ma , J. Wang , J. Liu , H. Qing , L. Song , L. Wang , Q. Hou , et al., Nat. Microbiol. 2020, 5, 1565. 32958858 10.1038/s41564-020-00790-y

[16] R. O. Rocha , C. Elowsky , N. T. T. Pham , R. A. Wilson , Nat. Microbiol. 2020, 5, 1472. 32929190 10.1038/s41564-020-0786-x

[17] M. Osés‐Ruiz , W. Sakulkoo , G. R. Littlejohn , M. Martin‐Urdiroz , N. J. Talbot , Proc. Natl. Acad. Sci. USA 2017, 114, E237. 28028232 10.1073/pnas.1611307114 PMC5240714

[18] M. Osés‐Ruiz , N. J. Talbot , Commun. Integr. Biol. 2017, 10, 1372067. 10.1080/19420889.2017.1372067 PMC5731507 29259729

[19] D. G. Saunders , Y. F. Dagdas , N. J. Talbot , Plant Cell 2010, 22, 2417. 20639448 10.1105/tpc.110.074492 PMC2929119

[20] J. C. Saldivar , S. Hamperl , M. J. Bocek , M. Chung , T. E. Bass , F. Cisneros‐Soberanis , K. Samejima , L. Xie , J. R. Paulson , W. C. Earnshaw , D. Cortez , T. Meyer , K. A. Cimprich , Science 2018, 361, 806. 30139873 10.1126/science.aap9346 PMC6365305

[21] Y. Xiao , L. Liu , T. Zhang , R. Zhou , Y. Ren , X. Li , H. Shu , W. Ye , X. Zheng , Z. Zhang , H. Zhang , Environ. Microbiol. 2021, 23, 774. 32431008 10.1111/1462-2920.15088

[22] H. Zhang , Q. Zhao , X. Guo , M. Guo , Z. Qi , W. Tang , Y. Dong , W. Ye , X. Zheng , P. Wang , Z. Zhang , Mol. Plant Microbe. Interact. 2014, 27, 446. 24405033 10.1094/MPMI-09-13-0271-R

[23] L. S. Ryder , Y. F. Dagdas , T. A. Mentlak , M. J. Kershaw , C. R. Thornton , M. Schuster , J. Chen , Z. Wang , N. J. Talbot , Proc. Natl. Acad. Sci. USA 2013, 110, 3179. 23382235 10.1073/pnas.1217470110 PMC3581893

[24] Z. Qi , Q. Wang , X. Dou , W. Wang , Q. Zhao , R. Lv , H. Zhang , X. Zheng , P. Wang , Z. Zhang , Mol. Plant Pathol. 2012, 13, 677. 22321443 10.1111/j.1364-3703.2011.00779.x PMC3355222

[25] D. G. Saunders , S. J. Aves , N. J. Talbot , Plant Cell 2010b, 22, 497. 20190078 10.1105/tpc.109.072447 PMC2845407

[26] T. K. Fung , R. Y. Poon , Semin. Cell Dev. Biol. 2005, 16, 335. 15840442 10.1016/j.semcdb.2005.02.014

[27] A. Koch , M. Wassenegger , New Phytol. 2021, 231, 54. 33774815 10.1111/nph.17364

[28] Y. Ning , W. Liu , G. L. Wang , Trends Plant Sci. 2017, 22, 1069. 29037452 10.1016/j.tplants.2017.09.010

[29] M. L. Gullino , P. Leroux , C. M. Smith , Crop Prot. 2000, 19, 1.

[30] N. Dulal , A. Rogers , Y. Wang , M. Egan , Fungal Genet. Biol. 2020, 140, 103385. 32305452 10.1016/j.fgb.2020.103385

[31] O. I. Aruoma , M. Grootveld , T. Bahorun , BioFactors 2006, 27, 1. 17012759 10.1002/biof.5520270101

[32] N. Tuteja , M. B. Singh , M. K. Misra , P. L. Bhalla , R. Tuteja , Crit. Rev. Biochem. Mol. Biol. 2001, 36, 337. 11563486 10.1080/20014091074219

[33] S. Dorsey , S. Tollis , J. Cheng , L. Black , S. Notley , M. Tyers , C. A. Royer , Cell Syst. 2018, 6, 539. 29792825 10.1016/j.cels.2018.04.012

[34] C. Koch , T. Moll , M. Neuberg , H. Ahorn , K. Nasmyth , Science 1993, 261, 1551. 8372350 10.1126/science.8372350

[35] M. Kõivomägi , M. P. Swaffer , J. J. Turner , G. Marinov , J. M. Skotheim , Science 2021, 374, 347. 34648313 10.1126/science.aba5186 PMC8608368

[36] F. Ferrezuelo , N. Colomina , B. Futcher , M. Aldea , Genome Biol. 2010, 11, R67. 20573214 10.1186/gb-2010-11-6-r67 PMC2911115

[37] J. Xiao , J. J. Turner , M. Kõivomägi , J. M. Skotheim , Curr. Biol. 2024, 34, 2434. 38749424 10.1016/j.cub.2024.04.052 PMC11247822

[38] M. J. Flynn , J. A. Benanti , Proc. Natl. Acad. Sci. USA 2022, 119, 2202469119.

[39] M. Pilkinton , R. Sandoval , J. Song , S. A. Ness , O. R. Colamonici , J. Biol. Chem. 2007, 282, 168. 17098733 10.1074/jbc.M609924200

[40] A. Kunova , C. Pizzatti , M. Bonaldi , P. Cortesi , Plant Dis. 2014, 98, 512. 30708720 10.1094/PDIS-04-13-0432-RE

[41] K. Vasudevan , C. M. V. Cruz , W. Gruissem , N. K. Bhullar , Front. Plant Sci. 2014, 505, 1. 10.3389/fpls.2014.00505 PMC4183131 25324853

[42] X. Y. Wu , B. Dong , X. M. Zhu , Y. Y. Cai , L. Li , J. P. Lu , B. Yu , J. L. Cheng , F. Xu , J. D. Bao , Y. Wang , X. H. Liu , F.‐C. Lin , Plant Commun. 2024, 5, 100724. 37771153 10.1016/j.xplc.2023.100724 PMC10873891

[43] M. J. BeltráelGarcíar , F. M. Prado , M. S. Oliveira , D. Ortiz‐Mendoza , A. C. Scalfo , A. Pessoa Jr , M. H. G. Medeiros , J. F. White , P. Di Mascio , PLoS One 2014, 9, 91616. 10.1371/journal.pone.0091616 PMC3960117 24646830

[44] C. P. Woloshuk , H. D. Sisler , M. C. Tokousbalides , S. R. Dutky , Pestic. Biochem. Phys. 1980, 14, 256.

[45] T. Zhang , X. Wang , X. Li , Y.‐N. Li , Y. Li , S. Wu , L. Xu , R. Zhou , J. Yang , G. Li , X. Liu , X. Zheng , Z. Zhang , H. Zhang , Plant Commun. 2023b, 4, 100561. 36774535 10.1016/j.xplc.2023.100561 PMC10363509

[46] J. Hu , M. Liu , A. Zhang , Y. Dai , W. Chen , F. Chen , W. Wang , D. Shen , M. J. Telebanco‐Yanoria , B. Ren , H. Zhang , H. Zhou , B. Zhou , P. Wang , Z. Zhang , Mol. Plant 2022, 15, 1347. 35799449 10.1016/j.molp.2022.07.001 PMC11163382

[47] P. Gan , R. Hiroyama , A. Tsushima , S. Masuda , A. Shibata , A. Ueno , N. Kumakura , M. Narusaka , T. X. Hoat , Y. Narusaka , Y. Takano , K. Shirasu , Environ. Microbiol. 2021, 23, 6004. 33780109 10.1111/1462-2920.15490

[48] H. Zhang , B. Li , Q. Fang , Y. Li , X. Zheng , Z. Zhang , Mol. Plant Pathol. 2016, 17, 108. 25880818 10.1111/mpp.12267 PMC6638462

[49] X. Liu , J. Yang , B. Qian , Y. Cai , X. Zou , H. Zhang , X. Zheng , P. Wang , Z. Zhang , PLoS Pathog. 2018, 14, 1007016. 10.1371/journal.ppat.1007016 PMC5933821 29684060

[50] K. S. Bruno , F. Tenjo , L. Li , J. E. Hamer , J. R. Xu , Eukaryotic Cell 2004, 3, 1525. 15590826 10.1128/EC.3.6.1525-1532.2004 PMC539019

[51] K. Zhong , X. Li , X. Le , X. Kong , H. Zhang , X. Zheng , P. Wang , Z. Zhang , PLoS Pathog. 2016, 12, 1005823. 10.1371/journal.ppat.1005823 PMC4996533 27556292

[52] K. Yonehara , N. Kumakura , T. Motoyama , N. Ishihama , J. F. Dallery , R. O'Connell , K. Shirasu , Mol. Plant Pathol. 2023, 24, 1451. 37522511 10.1111/mpp.13378 PMC10576178

[53] B. Li , X. Dong , R. Zhao , R. Kou , X. Zheng , H. Zhang , PLoS Pathog. 2019, 15, 1007754. 10.1371/journal.ppat.1007754 PMC6527245 31067272

[54] Z. Yin , C. Chen , J. Yang , W. Feng , X. Liu , R. Zuo , J. Wang , L. Yang , K. Zhong , C. Gao , H. Zhang , X. Zheng , P. Wang , Z. Zhang , Autophagy 2019, 15, 1234. 30776962 10.1080/15548627.2019.1580104 PMC6613890

[55] H. Zhang , K. Liu , X. Zhang , W. Song , Q. Zhao , Y. Dong , M. Guo , X. Zheng , Z. Zhang , Curr. Genet. 2010, 56, 517. 20848286 10.1007/s00294-010-0319-x

[56] J. Zhang , H. Li , W. Gu , K. Zhang , X. Liu , M. Liu , L. Yang , G. Li , Z. Zhang , H. Zhang , mBio 2023, 14, 0238123. 10.1128/mbio.02381-23 PMC10746245 37966176

[57] R. Yu , X. Shen , M. Liu , X. Liu , Z. Yin , X. Li , W. Feng , J. Hu , H. Zhang , X. Zheng , P. Wang , Z. Zhang , PLoS Pathog. 2021, 17, 1009657. 10.1371/journal.ppat.1009657 PMC8208561 34133468

[58] K. J. Livak , T. D. Schmittgen , Methods 2001, 25, 402. 11846609 10.1006/meth.2001.1262

[59] X. Liu , Y. Gao , Z. Guo , N. Wang , A. Wegner , J. Wang , X. Zou , J. Hu , M. Liu , H. Zhang , X. Zheng , P. Wang , U. Schaffrath , Z. Zhang , New Phytol. 2022, 235, 1163. 35451078 10.1111/nph.18169 PMC11164540

[60] J. Ma , L. Wei , K. Huang , D. Wang , J. Gao , X. Chen , H. Guo , S. Gao , M. Zhang , S. Li , C. Yu , J. Zhao , J. Wu , Q. Gu , S. T. Kim , R. Gupta , G. Xiong , C. Lo , Y. Liu , Y. Wang , New Phytol. 2025, 245, 1216. 39611538 10.1111/nph.20308

[61] J. Zhao , T. Long , Y. Wang , X. Tong , J. Tang , J. Li , H. Wang , L. Tang , Z. Li , Y. Shu , X. Liu , S. Li , H. Liu , J. Li , Y. Wu , J. Zhang , Plant Physiol. 2020, 182, 2047. 32029522 10.1104/pp.19.01487 PMC7140947

[62] D. M. Emms , S. Kelly , Mol. Biol. Evol. 2017, 34, 3267. 29029342 10.1093/molbev/msx259 PMC5850722

[63] H. Hu , W. He , Z. Qu , X. Dong , Z. Ren , M. Qin , H. Liu , L. Zheng , J. Huang , X. L. Chen , Adv. Sci. (Weinh) 2024, 11, 2403894. 38704696 10.1002/advs.202403894 PMC11234416

