# Supplementary material for: MoMtg1 Acts as a Novel Transcriptional Repressor of MoSwi6 During Appressorium‐Mediated Penetration in the Rice Blast Fungus
Source: Adv Sci (Weinh). 2025 Aug 28;12(43):e09002. doi: 10.1002/advs.202509002 (PMC12631829; doi:10.1002/advs.202509002)
Supplement: Supplementary file 1 — Supporting Information [file ADVS-12-e09002-s001.docx]

**
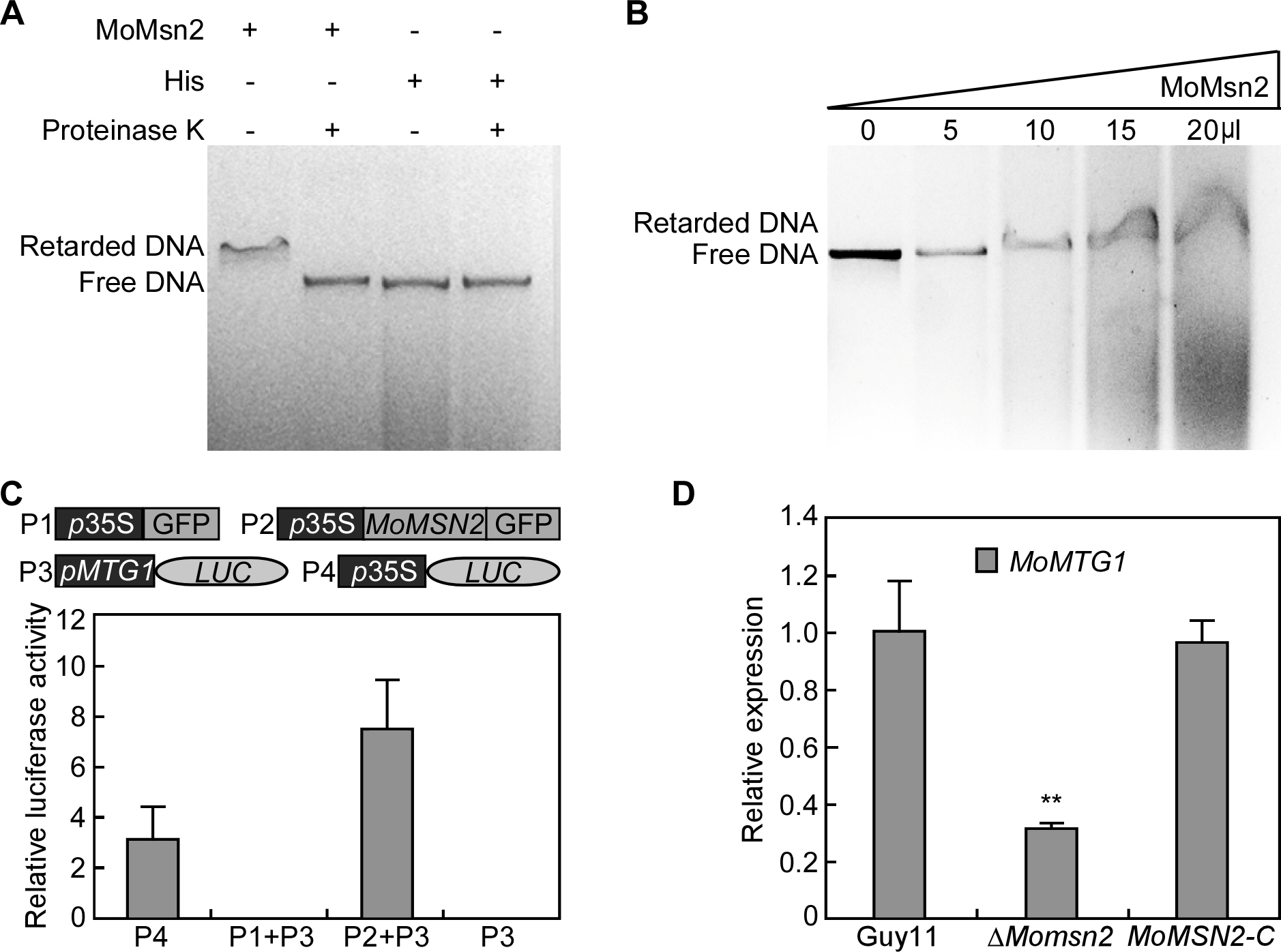
Figure S1. *MoMTG1* is a target gene of the transcription factor MoMsn2.** (A) Electrophoretic mobility shift assay (EMSA) validates the binding of MoMsn2 with the putative promoter sequence of *MoMTG1*. The 1500 bp DNA fragment of the putative promoter sequence of *MoMTG1* was incubated in the absence or presence of purified MoMsn2 protein. pET-32a-His empty protein or proteinase K was added after the incubation of MoMsn2 protein with DNA fragment as control. DNA-protein complexes were separated by 1.5% agarose gel electrophoresis and photographed. (B) EMSA assays with increased amounts of MoMsn2 protein. (C) Luciferase activity assay. The tested constructs were co-expressed or separately expressed in *N. benthamiana*. Luciferase activity was detected after 48 hpi. P1 and P3: negative control; P4: positive control. *p*35S: 35S promoter; *pMTG1*: *MoMTG1* promoter. GFP: green fluorescent protein; *LUC*: luciferase encoding gene. (D) Quantitative RT-PCR analyses the expression level of *MoMTG1* in the Δ*Momsn2* mutant. Error bars are standard deviations from three biological replicates and asterisks indicate statistically significant differences (*p*<0.01, two-tailed Student's *t*-test).

**

**

**Figure S2. Targeted deletion of *MoMTG1* in *M. oryzae*.** (A) Schematic diagram of replacement strategy of *MoMTG1* in *M. oryzae* genomic DNA. (B) Southern blot analyses the gene knockout mutant using *MoMTG1* (Probe 1) and *HPH* (Probe 2) probe, respectively.

**
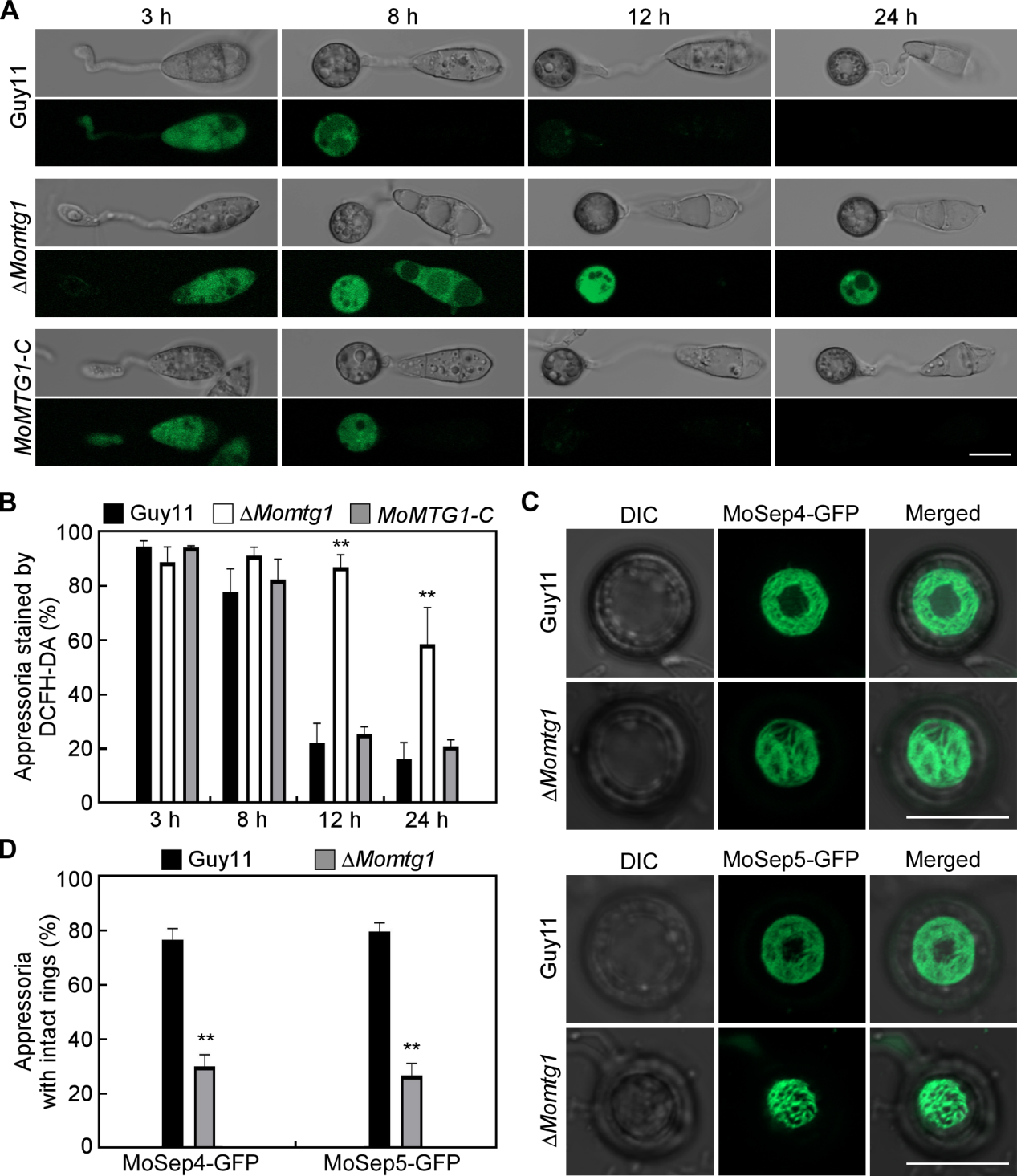
Figure S3. MoMtg1 is involved in the regulation of appressorium ROS accumulation and septin assembly.** (A) Appressorium was stained by DCFH-DA at different time points and observed under a fluorescence confocal microscopy. Bar=10 μm. (B) Statistical analysis of the appressoria stained by DCFH-DA. Error bars are standard deviations from three biological replicates with 100 conidia counted per replicate, and asterisks represent significant differences (*p*<0.01, two-tailed Student's *t*-test). (C) Appressorium septin rings were observed under a fluorescence confocal microscopy. Bar=10 μm. (D) Statistical analyses the appressoria with intact septin rings. Error bars are standard deviations from three biological replicates with 100 appressoria counted per replicate, and asterisks indicate statistically significant differences (*p*<0.01, two-tailed Student's *t*-test).

**
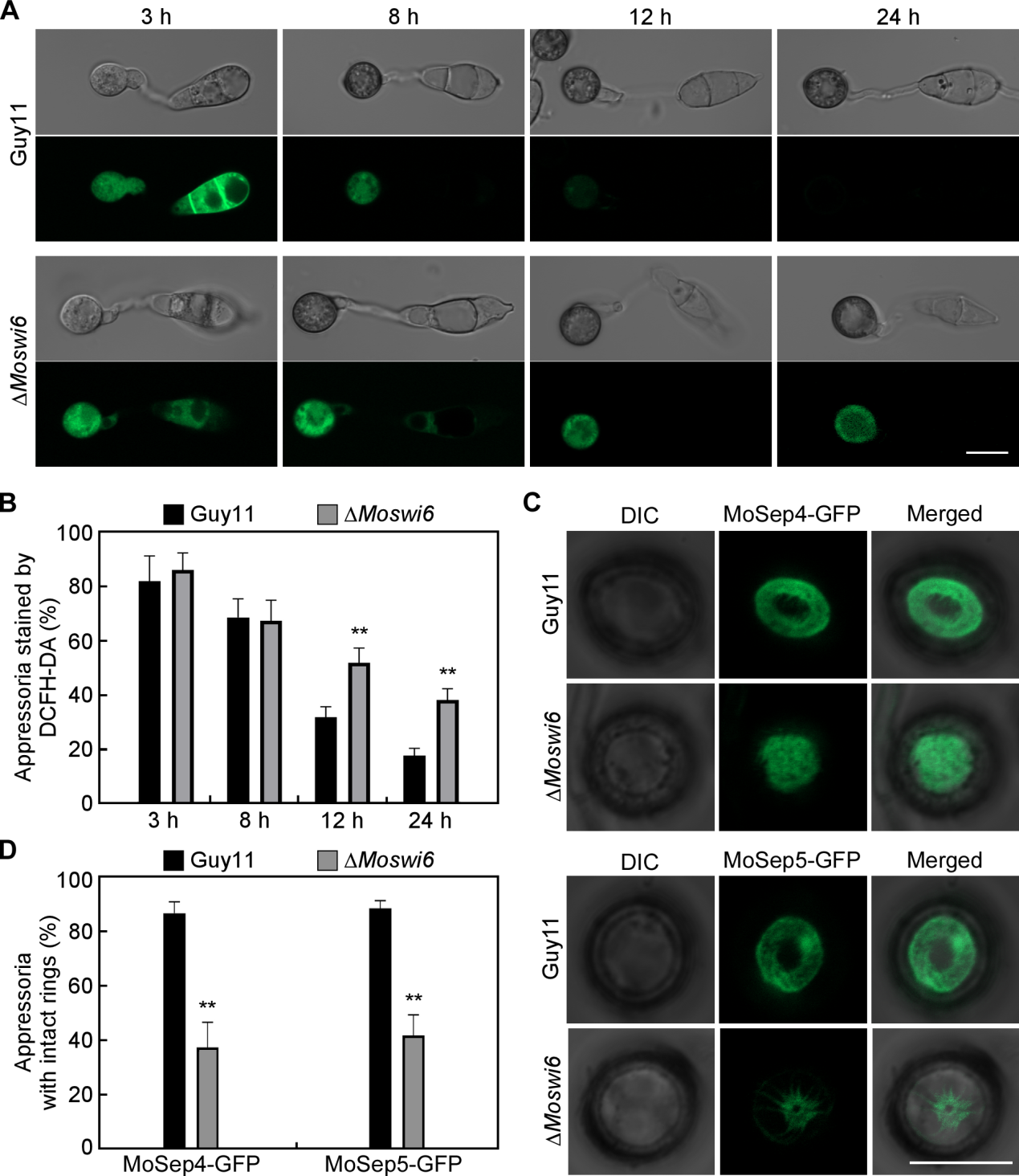
Figure S4. MoSwi6 is involved in the regulation of appressorium ROS accumulation and septin assembly.** (A) Appressorium was stained by DCFH-DA at different time points and observed under a fluorescence confocal microscopy. Bar=10 μm. (B) Statistical analysis of the appressoria stained by DCFH-DA. Error bars are standard deviations from three biological replicates with 100 conidia counted per replicate, and asterisks indicate significant differences (*p*<0.01, two-tailed Student's *t*-test). (C) Appressorium septin rings were observed under a fluorescence confocal microscopy. Bar=10 μm. (D) Statistical analyses the appressoria with intact septin rings. Error bars are standard deviations from three biological replicates with 100 appressoria counted per replicate, and asterisks indicate statistically significant differences (*p*<0.01, two-tailed Student's *t*-test).


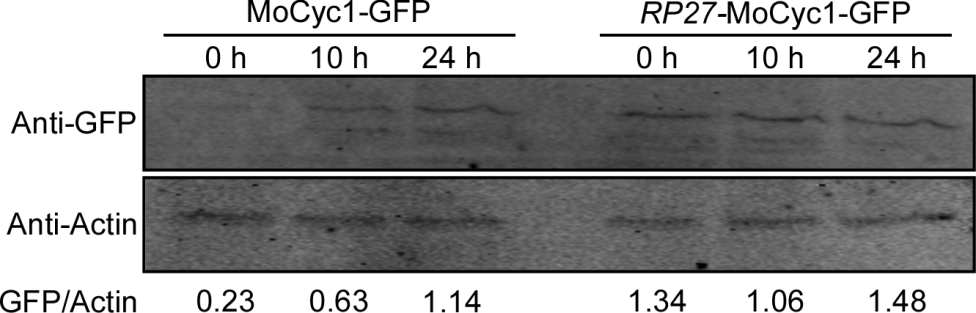


**Figure S5. *MoCYC1* overexpression results in increased accumulation of MoCyc1 during appressorium development.** Conidia of the tested strains were allowed to germinate on onion epidermis and collected at 0, 10 and 24 hpi. Each time point was subjected to analyze MoCyc1 protein abundance by Western blot with anti-GFP antibody. Actin was used as the loading control. *RP27*: enhanced promoter.


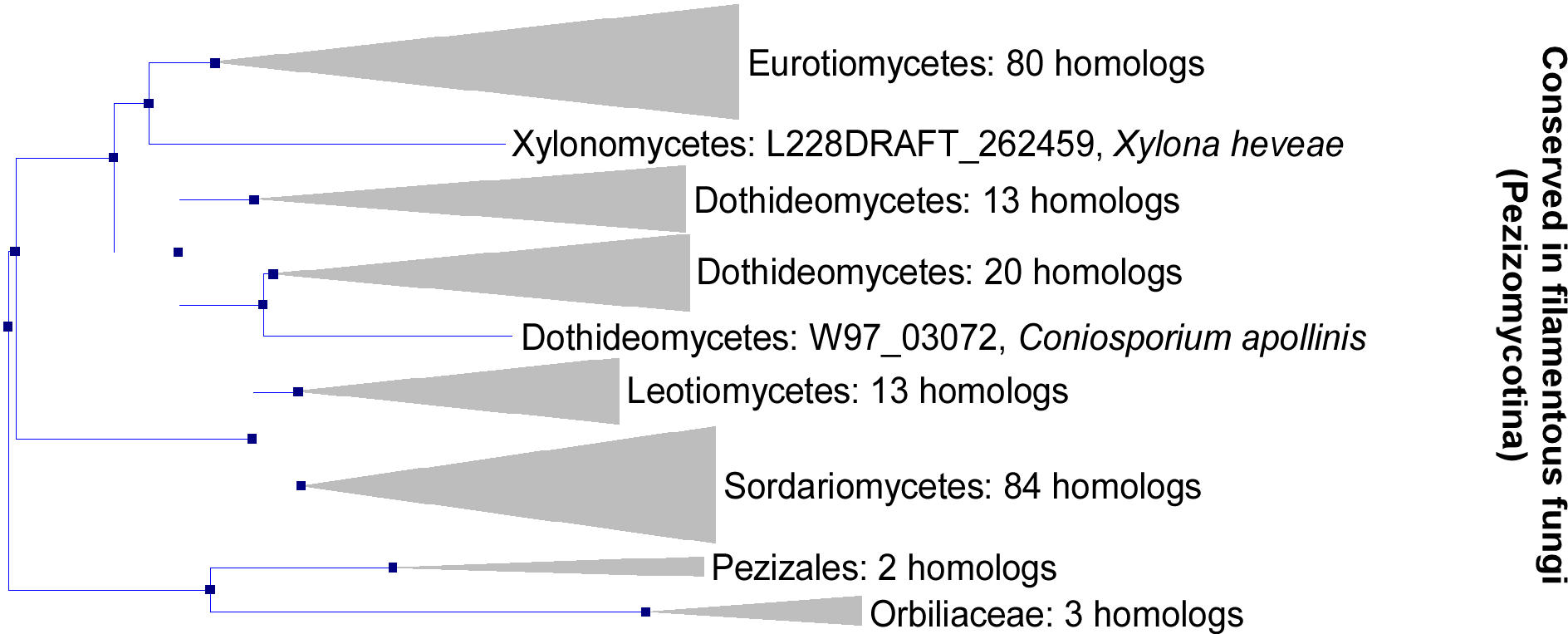


**Figure S6. Mtg1 is conserved in filamentous fungi.** The phylogenetic tree of Mtg1 proteins in different fungal lineages from Ensembl Fungi.

**
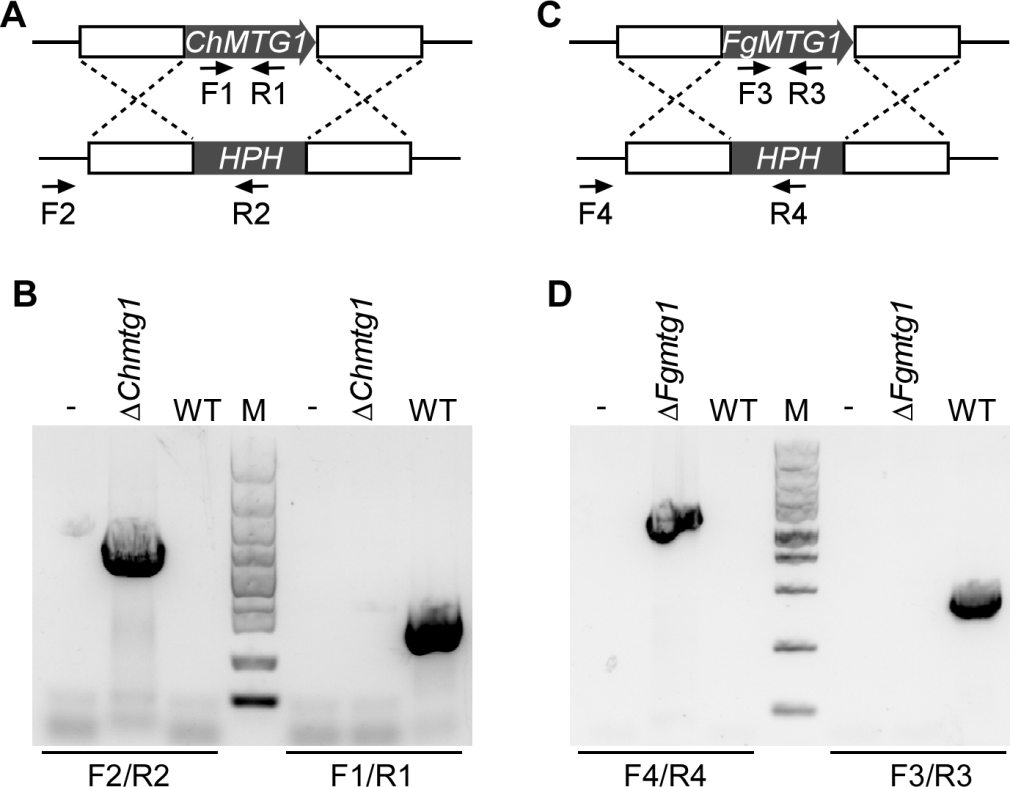
**

**Figure S7. Targeted deletion of *MoMTG1* in *M. oryzae*.** (A and C) Schematic diagram of replacement strategy of *ChMTG1* and *FgMTG1* in *C. higginsianum* and *F. graminearum* genomic DNA, respectively. (B and D) PCR analyses the gene knockout mutant using corresponding primers. M, DL5000 DNA marker; -, negative control.

Table S1. Phenotypic analysis of the Guy11, ∆*Momtg1* and *MoMTG1-C* strains.

| Strain | Growth (cm)^a^ | | Conidiation^b^  (🞨10^4^/cm^2^) | Abnormal conidia (%)^c^ | Conidial germination^d^  (%) | Appressorial formation^e^  (%) |
| --- | --- | --- | --- | --- | --- | --- |
|  | CM | MM |  |  |  |  |
| Guy11 | 5.2±0.1 | 4.9±0.1 | 11.5±0.2 | 12.0±1.5 | 98.0±1.5 | 99.0±0.4 |
| ∆*Momtg1* | 4.1±0.2** | 3.2±0.1** | 0.26±0.25** | 75.2±1.3** | 97.6±1.1 | 98.0±0.3 |
| *MoMTG1-C* | 5.0±0.1 | 4.6±0.1 | 11.5±0.5 | 14.8±1.2 | 98.2±1.6 | 99.0±0.5 |

±SD was calculated from three repeated experiments and asterisks indicate statistically significant differences (*p*<0.01, two-tailed Student's *t*-test).

^a^Colony diameter after cultured at 28°C for 7 days.

^b^Conidial number of the indicated strains.

^c^Percentage of abnormal conidia, (*n*=3 experiments, conidia=100).

^d^Percentage of conidial germination at 24 hpi, (*n*=3 experiments, conidia=100).

^e^Percentage of appressorium formation at 24 hpi, (*n*=3 experiments, conidia=100).

Table S2. Primers used in this study.

| Primer name | Sequence （5’-3’） | Remark |
| --- | --- | --- |
| *MoMTG1*ko-LF-1 | CACTCACGATATCCTGTCCAG | Amplification of *MoMTG1* 5’ flank sequence |
| *MoMTG1*ko-LF-2 | AAATAGGCATTGATGTGTTGACCTCCGGTCTAAAGAAGTAAAAGAGGG | Amplification of *MoMTG1* 5’ flank sequence |
| *MoMTG1*ko-RF-2 | AGCACTCGTCCGAGGGCAAAGGAATAGAGACGAATCAAACCCCATGCTCC | Amplification of *MoMTG1* 3’ flank sequence |
| *MoMTG1*ko-RF-1 | CTGGAGAGTGAGAGATTGGAT | Amplification of *MoMTG1* 3’ flank sequence |
| HPH-F | GGAGGTCAACACATCAATGCC | Amplification of *HPH* probe sequence |
| HPH-R | CTCTATTCCTTTGCCCTCGGA | Amplification of *HPH* probe sequence |
| *MoMTG1*ko-F | TGAGGCTGCTTATCGTTCCG | Amplification of *MoMTG1* probe sequence |
| *MoMTG1*ko-R | CTTTGGGTCGCTGGTAGTGT | Amplification of *MoMTG1* probe sequence |
| pYF11-*MoMTG1C*-F | GGCGAATTGGGTACTCAAATTGGTTCGTGACAGCTCGAAGTCGCT | *MoMTG1* complementation (*with GFP tag*) |
| pYF11-*MoMTG1C*-R | ACAGCTCCTCGCCCTTGCTCACCGTGCCAGTCATCTTGACAT | *MoMTG1* complementation (*with GFP tag*) |
| *FgMTG1*ko-LF-1 | CTGTTGCAGAACTACTCCTG | Amplification of *FgMTG1* 5’ flank sequence |
| *FgMTG1*ko-LF-2 | GCATTGATGTGTTGACCTCCGACAGCTGAAGTGGTTGCAG | Amplification of *FgMTG1* 5’ flank sequence |
| *FgMTG1*ko-RF-1 | CGACAGCCTCAAAACTAACG | Amplification of *FgMTG1* 3’ flank sequence |
| *FgMTG1*ko-RF-2 | CCGAGGGCAAAGGAATAGAGTCTCACTTCAATGGTGGCCT | Amplification of *FgMTG1* 3’ flank sequence |
| *FgMTG1*ko-BW | GACTGGCTTTGCTTACTGGT | Validation of *FgMTG1* deletion transformants |
| HphYZRR | CGCTACTGCTACAAGTGGGGCT | Validation of *MTG1* deletion transformants |
| *FgMTG1*ko-P1 | ATGCTGTTGCGCTTTCTGCCCT | Validation of *FgMTG1* deletion transformants |
| *FgMTG1*ko-P2 | TAACTTGGGGCCCACAGGTT | Validation of *FgMTG1* deletion transformants |
| pYF11-*FgMTG1C*-F | TATAGGGCGAATTGGGTACTCAAATTGGTTTGGAAAGCGAGCCATGGCAA | *FgMTG1* complementation (*with GFP tag*) |
| pYF11-*FgMTG1C*-R | CCCGGTGAACAGCTCCTCGCCCTTGCTCACAGTATCAGCCATCTTGACGT | *FgMTG1* complementation (*with GFP tag*) |
| *ChMTG1*ko-LF-1 | GTTCCCATCTTGTTACAGGG | Amplification of *ChMTG1* 5’ flank sequence |
| *ChMTG1*ko-LF-2 | GGTGTCGTGAAGGTCCCGGACAA | Amplification of *ChMTG1* 5’ flank sequence |
| *ChMTG1*ko-RF-1 | CTAGAACTGCTTGGCTAGCG | Amplification of *ChMTG1* 3’ flank sequence |
| *ChMTG1*ko-RF-2 | AGCATTTTTGACCAGCGAAT | Amplification of *ChMTG1* 3’ flank sequence |
| *ChMTG1*ko-BW | CCAAAAGCTCCAACTATCCG | Validation of *ChMTG1* deletion transformants |
| *ChMTG1*ko-P1 | TCAAGCGAAGCCATTGACTT | Validation of *ChMTG1* deletion transformants |
| *ChMTG1*ko-P2 | ATAGTGGTTGGGGGTCTGCG | Validation of *ChMTG1* deletion transformants |
| pYF11-*ChMTG1C*-F | GGCGAATTGGGTACTCAAATTGGTTACAATTGTCTTTGGCCTGCT | *ChMTG1* complementation (*with GFP tag*) |
| pYF11-*ChMTG1C*-R | TGAACAGCTCCTCGCCCTTGCTCACTGCATCAGCCATCTTGACGT | *ChMTG1* complementation (*with GFP tag*) |
| SUMO-MoMtg1-F | TGAGAATCTTTATTTTCAGGGCGCCATGTTTGGAGGGTTGCACT | Construction of  pETSUMO-*MoMTG1* vector |
| SUMO-MoMtg1-R | GTGGTGGTGGTGGTGCTCGAGTGCGGCTTACGTGCCAGTCATCTTGA | Construction of  pETSUMO-*MoMTG1* vector |
| GST-MoSwi6-F | GGATCTGGTTCCGCGTGGATCCATGGCGTCGACGGTCGCC | Construction of  pGEX-4T-2-*MoSWI6* vector |
| GST-MoSwi6-R | CTCGAGTCGACCCGGGAATTCTCACCCGATAACCCCTTCTA | Construction of  pGEX-4T-2-*MoSWI6* vector |
| BD-MoMtg1-F | TCTCAGAGGAGGACCTGCATATGTTTGGAGGGTTGCACT | Construction of  pGBKT7-*MoMTG1* vector |
| BD-MoMtg1-R | AGGGGTTATGCTAGTTATGCTTACGTGCCAGTCATCTTGA | Construction of  pGBKT7-*MoMTG1* vector |
| AD-MoSwi6-F | ATACGACGTACCAGATTACGCTCATATGGCGTCGACGGTCGCC | Construction of  pGADT7-*MoSWI6* vector |
| AD-MoSwi6-R | ATCGATGCCCACCCGGGTGGAATTCTCACCCGATAACCCCTTCTA | Construction of  pGADT7-*MoSWI6* vector |
| pHZ126-MoSwi6-F | GGCGAATTGGGTACTCAAATTGGTTCTATCCAACCAGTCGTACGG | Construction of pHZ126-MoSwi6 vector (*with 3×Flag tag*) |
| pHZ126-MoSwi6-R | AATCACCGTCATGGTCTTTGTAGTCCCCGATAACCCCTTCTACAC | Construction of pHZ126-*MoSWI6* vector (*with 3×Flag tag*) |
| RP27-*MoCYC1*-F | TTTCGTAGGAACCCAATCTTCAAAATGCCCCCAGTAAGTCAGAA | Construction of pYF11*-MoCYC1* vector (*with GFP tag, RP27* promoter) |
| RP27-*MoCYC1*-R | TGAACAGCTCCTCGCCCTTGCTCACCAGCTGGTCGATAGCTACGT | Construction of pYF11*-MoCYC1* vector (*with GFP tag, RP27* promoter) |
| *MoCYC1Pro*-F | GTATCAAAAGACACGCGCAG | Amplification of *MoCYC1* promoter |
| *MoCYC1Pro*-R | AGTGGCCAAGGAGGATTTTT | Amplification of *MoCYC1* promoter |
| *MoCYC1Pro*-AF | GTATCAAAAGACACGCGCAG | Amplification of  *MoCYC1*^Δ^*^WCGCGTY^* promoter |
| *MoCYC1Pro*-AR | CTGAGGTTGTTGAAATAGCG | Amplification of  *MoCYC1^ΔWCGCGTY^* promoter |
| *MoCYC1Pro*-BF | CGCTATTTCAACAACCTCAGCCTCGTGCGCGACCCTGGAATTAAG | Amplification of  *MoCYC1*^Δ^*^WCGCGTY^* promoter |
| *MoCYC1Pro*-BR | AAATGCAGGAGACGTGCGTC | Amplification of  *MoCYC1*^Δ^*^WCGCGTY^* promoter |
| *MoCYC1Pro*-CF | GACGCACGTCTCCTGCATTTAATAACCACAAAAGCTCGCG | Amplification of  *MoCYC1*^Δ^*^WCGCGTY^* promoter |
| *MoCYC1Pro*-CR | TGCCCTAGCCGGCGTGTCACAGTCGATGGCGGAACAAGGC | Amplification of  *MoCYC1*^Δ^*^WCGCGTY^* promoter |
| *MoCYC1Pro*-DF | GTGACACGCCGGCTA | Amplification of  *MoCYC1*^Δ^*^WCGCGTY^* promoter |
| *MoCYC1Pro*-DR | AGTGGCCAAGGAGGATTTTT | Amplification of  *MoCYC1*^Δ^*^WCGCGTY^* promoter |
| 0800-*MoCYC1Pro*-F | CTCACTATAGGGCGAATTGGGTACGTATCAAAAGACACGCGCAG | Construction of 0800-*MoCYC1Pro* vector |
| 0800-*MoCYC1Pro*-R | TCTTTATGTTTTTGGCGTCTTCCATAGTGGCCAAGGAGGATTTTT | Construction of 0800-*MoCYC1Pro* vector |
| pBIN-MoSwi6-F | AACGATAGCCGGTACCCCCGGGATGGCGTCGACGGTCGCC | Construction of pBIN-*MoSWI6* vector (*with GFP tag*) |
| pBIN-MoSwi6-R | ACAGCTCCTCGCCCTTGCTCACCATCCCGATAACCCCTTCTACAC | Construction of pBIN-*MoSWI6* vector (*with GFP tag*) |
| pBIN-MoMtg1-F | AACGATAGCCGGTACCCCCGGGATGTTTGGAGGGTTGCAC | Construction of pBIN-*MoMTG1* vector (*with GFP tag*) |
| pBIN-MoMtg1-R | ACAGCTCCTCGCCCTTGCTCACCATCGTGCCAGTCATCTTGACAT | Construction of pBIN- *MoMTG1* vector (*with GFP tag*) |
| *MoCYC1Pro*-F1 | AAGAGCTAGGCACGAAAGGC | ChIP-qPCR analysis of *MoCYC1* promoter |
| *MoCYC1Pro*-R1 | GCAGTAACGGCCAAGCAA | ChIP-qPCR analysis of *MoCYC1* promoter |
| *MoCYC1Pro*-F2 | TCTGTCCCGGTGATCAGCTA | ChIP-qPCR analysis of *MoCYC1* promoter |
| *MoCYC1Pro*-R2 | TAGACGTGTGTGAATCGAGA | ChIP-qPCR analysis of *MoCYC1* promoter |
| *MoMTG1*-qF | ATGAAGCCTAAACCCGGACA | qRT-PCR analysis of *MoMTG1* gene |
| *MoMTG1*-qR | TTTGCTTGTCCACTGCCTTC | qRT-PCR analysis of *MoMTG1* gene |
| *MoCYC1*-qF | AAACAAAGAAGGGGGTTGCT | qRT-PCR analysis of *MoCYC1* gene |
| *MoCYC1*-qR | CGTTTCCATTTGTGACGTTG | qRT-PCR analysis of *MoCYC1* gene |
| FL4362ActinF | CCATGTACCCTGGTCTTTCG | qRT-PCR analysis of *ACTIN* gene |
| FL4362ActinR | TTCGAGATCCACATCTGCTG | qRT-PCR analysis of *ACTIN* gene |
| *MoRSY1*-qF | CGACTCCAAGGACTGGGATA | qRT-PCR analysis of *MoRSY1* gene |
| *MoRSY1*-qR | GTCCTCGGACACCTTCTCC | qRT-PCR analysis of *MoRSY1* gene |
| *MoBUF1*-qF | ACGCCGTCTACTCAGGATCA | qRT-PCR analysis of *MoBUF1* gene |
| *MoBUF1*-qR | TCTCGCCGTTTGGAATGTAT | qRT-PCR analysis of *MoBUF1* gene |
| *MoALB1*-qF | GCAATGTCGGTCCCAACTAC | qRT-PCR analysis of *MoALB1* gene |
| *MoALB1*-qR | ATCTCAAAGGCGATGACACC | qRT-PCR analysis of *MoALB1* gene |
| *28S rDNA LL* | TACGAGAGGAACCGCTCATTCAGATAATTA | qRT-PCR analysis of fungal biomass |
| *28S rDNA RR* | TCAGCAGATCGTAACGATAAAGCTACTC | qRT-PCR analysis of fungal biomass |
| *RUBQ1 LL* | GTGGTGGCCAGTAAGTCCTC | qRT-PCR analysis of fungal biomass |
| *RUBQ1 RR* | GGACACAATGATTAGGGATCA | qRT-PCR analysis of fungal biomass |
| *MTG1i*-sense-F | CCCTCGAGCCGCTTGTCAAGCTACTAC | Amplification of *MoMTG1* silencing sense sequence |
| *MTG1i*-sense-R | CCATCGATCTCGTACGAATTGCCCAATC | Amplification of *MoMTG1* silencing sense sequence |
| *MTG1i*-antisense-F | GGACTAGTCCGCTTGTCAAGCTACTACG | Amplification of *MoMTG1* silencing antisense sequence |
| *MTG1i*-antisense-R | CGGAATTCCTCGTACGAATTGCCCAATC | Amplification of *MoMTG1* silencing antisense sequence |
